# ScaffoldForge: Bioinspired generative design of 3D-printed bone scaffolds via conditional diffusion for large segmental bone defect reconstruction

**DOI:** 10.1371/journal.pone.0361041

**Published:** 2026-10-28

**Authors:** Yaohua Zhang, Hongyue Zhang, Huaqing Yang

**Affiliations:** Beijing Rehabilitation Hospital, Capital Medical University, Beijing, China; University of Padova: Universita degli Studi di Padova, ITALY

## Abstract

We present ScaffoldForge, a 3D conditional diffusion framework for the generative design of bone scaffolds. ScaffoldForge maps a set of standardized trabecular descriptors (bone volume fraction BV/TV, trabecular thickness Tb.Th, trabecular spacing Tb.Sp, trabecular number Tb.N, connectivity density Conn.D, structure model index SMI, degree of anisotropy DA, and permeability *k*) to a 3D scaffold architecture in a single forward pass. A multi-objective conditioning mechanism couples these descriptors through adaptive instance normalization, and a physics-constrained loss adds empirical penalties for stiffness, manufacturing feasibility, and perfusion, evaluated on the denoiser’s reconstruction of the clean scaffold so that the penalties carry gradient. To address the scarcity of paired geometry–property data, we describe a procedural pipeline whose generated scaffolds reproduce the morphometric distributions of real micro-CT references. In an *in silico* evaluation on three benchmark datasets, ScaffoldForge attains an estimated 56.6 MPa compressive strength (8.2% over topology optimization; paired *t*-test, *p* = 0.043, *n* = 5 seeds) and reduces design-time cost from 8,400–12 seconds per design, a 700× speedup. Generated structures are diverse rather than memorized, and morphometric accuracy holds across all seven shape descriptors. Three analyses probe the reliability of these results given the small training corpus. Equivalence testing replaces the earlier argument from non-significant difference tests: the procedural and real descriptor distributions are equivalent at a medium margin of 0.5 standard deviations for all seven descriptors, but the 50 available real reference volumes cannot establish equivalence at a strict 0.3-standard-deviation margin for five of them. A classifier two-sample test shows that generated scaffolds retain a measurable but shrinking signature of the procedural generator. Enlarging the corpus from 250 to 2,050 scaffolds leaves in-distribution accuracy nearly unchanged while reducing the cross-dataset descriptor error on unseen anatomy from 0.079 to 0.058, which localizes the cost of the small corpus in generalization rather than in fit. We further illustrate a CT-to-scaffold pipeline for personalized generation. All results are computational; physical, biological, manufacturing, and regulatory validation remain future work.

## 1 Introduction

Large segmental bone defects, defined as defects exceeding a critical size that prevents spontaneous healing, affect over 2 million patients annually worldwide and impose significant healthcare costs [1,2]. Autologous bone grafting, the current gold standard, suffers from donor site morbidity, limited graft availability, and inconsistent integration outcomes [3]. Allograft transplantation carries risks of immune rejection and disease transmission. Three-dimensional (3D) printed bone scaffolds address these limitations by enabling patient-specific, anatomically-shaped constructs with controlled microarchitectures [4,5], yet translating personalized scaffold designs into clinical practice requires satisfying multiple competing biological and mechanical objectives simultaneously. The gap between scaffold design capability and clinical need remains wide: current commercial scaffolds typically offer a limited set of geometric presets, while patients with irregular defect geometries and site-specific mechanical requirements would benefit from scaffolds generated on demand from imaging data [6].

The design space for load-bearing bone scaffolds is governed by conflicting constraints that resist simple optimization. Porosity must exceed 60% to permit vascularization and nutrient transport [7], while compressive strength should surpass 50 MPa for load-bearing applications [8]. Permeability needs to support nutrient diffusion without sacrificing structural walls, and biodegradation kinetics must match the 3-to-6-month bone regeneration timeline [5]. These objectives are inherently coupled: increasing porosity to improve vascularization reduces mechanical stiffness, and architectures tuned for permeability may violate additive manufacturing constraints such as minimum feature size and overhang limits [8]. The clinical context compounds this complexity: patient-specific defect geometries extracted from CT scans impose anatomical boundary conditions that further constrain the feasible design space [9]. This coupling makes brute-force parametric search over the design space computationally prohibitive, motivating computational approaches that can navigate these tradeoffs efficiently.

Topology optimization (TO) methods, particularly solid isotropic material with penalization (SIMP), find optimal material distributions by iteratively removing low-density elements under mechanical constraints [10]. While TO produces mechanically efficient structures, it requires 100–200 finite element analysis (FEA) evaluations per design and cannot directly include permeability or degradation objectives [11]. Multi-objective evolutionary algorithms such as NSGA-II address this limitation by simultaneously optimizing stiffness, porosity, and permeability [12], yet converge slowly (hours per design) and produce architectures that, although Pareto-optimal, are often irregular and difficult to manufacture. Data-driven surrogates such as 3D CNNs speed up property prediction of predefined TPMS families [13], but these predictors cannot propose novel topologies outside their training distribution. Bayesian optimization reduces evaluation count compared to evolutionary methods but still requires dozens of FEA simulations and lacks generative capability [14]. More recently, generative models have entered the scaffold design space: ScaffoldGAN [15] introduced adversarial networks for 3D scaffold synthesis but suffers from training instability and mode collapse, and Scaffold Diffusion [16] applies discrete diffusion to generate voxel-based structures without functional conditioning. No existing method generates diverse scaffold architectures conditioned on multiple biomechanical specifications in a single forward pass.

We present ScaffoldForge, a conditional diffusion model that addresses this gap by learning to generate scaffold architectures directly from functional specifications. Built on the denoising diffusion probabilistic model (DDPM) framework [17], ScaffoldForge encodes target bone volume fraction (BV/TV), trabecular thickness (Tb.Th), trabecular spacing (Tb.Sp), and permeability (*k*) through a shared MLP conditioner injected into the 3D U-Net backbone via adaptive instance normalization (AdaIN). Classifier-free guidance [18] strengthens conditioning at inference time, while a physics-constrained loss penalizes violations of stiffness bounds, manufacturing overhang limits, and minimum permeability during training. The forward diffusion process gradually adds noise to scaffold geometries over 1000 timesteps, and the reverse process learns to denoise conditioned on the specification vector, yielding a generative model that produces complete 3D architectures in approximately 12 seconds. Unlike the conditional latent diffusion approach of [19], which generates trabecular bone microstructures from morphological parameters, ScaffoldForge targets the scaffold design problem, where functional specifications extend beyond morphology to include explicitly constrained biomechanical and manufacturing objectives.

Two concurrent lines of work highlight the timeliness of our approach while underscoring its distinct contribution [20]. proposed TriTopo-LGDM, which integrates topology optimization with a latent graph diffusion model for trabecular bone scaffold reverse design; their method generates scaffold geometries on a graph representation initialized from TO solutions, meaning the generative model supplements rather than replaces iterative optimization [21]. introduced a peridynamics-driven conditional diffusion model that embeds physical constraints for fracture prediction in porous structures, demonstrating that physics-based loss terms improve structural fidelity, though their focus is on fracture prediction rather than scaffold synthesis [22]. proposed MicroLad for 3D microstructure reconstruction via latent diffusion and score distillation, targeting composite material design rather than biomedical scaffolds with multi-objective biomechanical constraints. ScaffoldForge differs from these approaches in three respects: it generates scaffolds from specifications alone (no TO initialization), conditions on a broad set of biomechanical targets rather than geometric priors, and penalizes manufacturing infeasibility through explicit loss terms.

The main contributions of this paper are:

- We design a multi-objective conditioning mechanism that jointly controls a broad set of standardized trabecular descriptors (BV/TV, Tb.Th, Tb.Sp, Tb.N, Conn.D, SMI, DA, and permeability) through learned AdaIN embeddings, rather than the three morphological descriptors used in prior conditional scaffold models (Section 3.4).
- We add a physics-constrained training objective with empirical penalties for stiffness, manufacturing overhang, and minimum permeability, evaluated on the denoiser’s reconstruction of the clean scaffold rather than on the ground-truth geometry, and we are explicit that these are soft constraints reflecting physical requirements rather than first-principles governing laws (Section 3.5).
- We document a procedural data-generation pipeline and report an evaluation with statistical testing, extended-descriptor accuracy, diversity and memorization analysis, and cross-dataset external validation (Section 4).
- We quantify how far the small training corpus limits the results, through equivalence testing against real micro-CT morphometry, a classifier two-sample test for residual simulator signature, a corpus-size and denoiser-capacity study, and a regularization ablation (Sections 4.10 and 4.11).

## 2 Related work

Our work relates to three lines of research: computational optimization for scaffold design, generative models for 3D structure synthesis, and physics-based conditional generation for engineering design.

### 2.1 Computational optimization for bone scaffold design

Topology optimization (TO) methods, particularly solid isotropic material with penalization (SIMP), find mechanically efficient material distributions by iteratively removing low-density elements under stiffness and volume constraints [10]. SIMP-based approaches have been adapted for scaffold design by incorporating permeability and biodegradation objectives alongside structural compliance [11], though each design requires 100–200 finite element analysis (FEA) evaluations, making patient-specific exploration prohibitively slow. Multi-objective evolutionary algorithms address this limitation by simultaneously optimizing stiffness, porosity, and permeability [12]. demonstrated that NSGA-II can identify Pareto-optimal scaffold architectures, and their subsequent work [8] simultaneously tuned stiffness, permeability, and surface area for metallic scaffolds, showing that stiffness and permeability are strongly conflicting objectives [23]. recently combined Haversian-system-inspired biomimetic architecture with NSGA-II optimization for femoral defect scaffolds, achieving yield strengths of 62–88 MPa but requiring hours per design. Bayesian optimization reduces evaluation count compared to evolutionary methods [14], yet still demands dozens of FEA simulations and lacks generative capability. Machine learning surrogates have been explored as faster property predictors: [24] used regression models to screen TPMS scaffold families [25], coupled neural networks with orthogonal-array-driven FEA for scaffold optimization, and [26] applied AI-tuned lattice structures to biomechanical scaffold design [13]. trained 3D CNNs to predict mechanical properties of predefined TPMS families but their predictor cannot propose novel topologies outside its training distribution. Reviewing this area [27], identify a persistent gap: existing computational methods tune within predefined parametric families (TPMS, lattice, Voronoi), which restricts the design space to geometrically regular structures that may not match patient-specific anatomical and biomechanical requirements [6,9]. ScaffoldForge addresses this gap by learning a generative mapping directly from functional specifications to scaffold geometries, bypassing iterative FEA entirely.

### 2.2 Generative models for 3D structure and volumetric design

Generative adversarial networks (GANs) [28] have been applied to 3D porous structure synthesis, with ScaffoldGAN [15] introducing adversarial training for bone scaffold generation. However, GAN-based approaches suffer from training instability, mode collapse, and limited control over generated properties, as [29] confirm in their comparison of DDPMs against GANs for 3D clay microstructure generation, finding that DDPMs produce structurally consistent realizations with higher fidelity than both Wasserstein GANs and progressive growing GANs. Variational autoencoders (VAEs) [30] enable stochastic generation but typically produce blurry, low-detail outputs due to the reconstruction-regularization tradeoff inherent in the evidence lower bound. The introduction of denoising diffusion probabilistic models (DDPMs) [17] overcame these limitations: by learning to reverse a gradual noising process, DDPMs generate high-fidelity samples with stable training objectives, and [31] demonstrated that diffusion models surpass GANs on image synthesis benchmarks when augmented with classifier guidance. Latent diffusion models [32] further improved efficiency by operating in a compressed latent space, enabling high-resolution generation with reduced computational cost. For volumetric data [33], extended diffusion to 3D point cloud generation, and [34] proposed 3DSinDDPM for single-volume porous microstructure reconstruction using conditional diffusion with an auxiliary loss for controllable generation. In the scaffold domain [16], applied discrete diffusion to generate sparse voxel-based structures but did not condition on biomechanical specifications, and [19] introduced conditional latent diffusion for trabecular bone microstructure synthesis conditioned on morphological parameters yet without manufacturing or stiffness constraints [35]. developed a diffusion-based generative framework for composite microstructure inverse design, combining conditional diffusion with a CNN surrogate for property prediction, validating the feasibility of diffusion-driven inverse design for engineering structures. These works demonstrate that diffusion models can handle 3D volumetric generation with stable training, but none jointly conditions on multiple biomechanical specifications with physics-based constraints, which is precisely the capability ScaffoldForge introduces.

### 2.3 Physics-based and conditional generation for engineering design

Physics-informed neural networks (PINNs) [36] embed governing equations as loss terms to constrain network predictions, a paradigm that has been extended from simulation to generative modeling. In structural design [37], trained a conditional diffusion model to map structural performance requirements to flexible support sketches for optical mirrors, achieving surface accuracy of 15 nm at room temperature, though their conditioning is limited to a single performance metric [38]. proposed Stress-Diff, a conditional diffusion model for stress field prediction in drilled flange connections, achieving 60× speedup over FEA with a variance-regularized loss and classifier-free guidance; while this demonstrates effective conditioning for structural predictions, it generates stress fields rather than structural geometries [39]. trained a biomechanics-conditioned diffusion model on IVOCT images to predict post-PCI plaque morphology, achieving structural similarity index above 0.89 and demonstrating that physics-based conditioning improves generation fidelity in biomedical contexts, though their model generates deformations of existing structures rather than novel designs. For inverse design of bone scaffolds in particular, concurrent work has emerged: [20] proposed TriTopo-LGDM, which integrates topology optimization with a latent graph diffusion model for trabecular bone reverse design, initializing generation from TO solutions; [21] introduced a peridynamics-driven conditional diffusion model for fracture prediction in porous structures, embedding physical constraints via a two-stage architecture; and [22] proposed MicroLad for 3D microstructure reconstruction via latent diffusion and score distillation targeting composite material design [40]. demonstrated data-driven inverse design of bicontinuous multiscale structures for bone implants using deep generative models, achieving tunable mechanical properties across multiple scales. These methods either rely on TO initialization (TriTopo-LGDM), target fracture prediction rather than scaffold synthesis (peridynamics diffusion), or focus on composite material design without biomechanical constraints (MicroLad). ScaffoldForge differs from these works by generating scaffold architectures from functional specifications alone, conditioning on a comprehensive set of biomechanical targets (stiffness, porosity, permeability) through adaptive instance normalization [41] and classifier-free guidance [18], and enforcing manufacturing feasibility through explicit physics-constrained loss terms.

## 3 Materials and methods

Table 1 summarizes the key notations used throughout this section.

**Table 1:** Summary of notations used in this paper.

Notation	Description
**x**_0_	Clean scaffold geometry, ${𝐱}_{0}\in \{0,1{\}}^{64\times 64\times 64}$
${𝐱}_{t}$	Noisy scaffold at diffusion timestep *t*
${\hat{𝐱}}_{0}({𝐱}_{t},t,𝐜)$	Denoiser’s reconstruction of the clean scaffold from ${𝐱}_{t}$
s(·)	Relaxed occupancy field, $s({\hat{𝐱}}_{0})\in [0,1{]}^{N^{3}}$
κ,τ	Sharpness and threshold of the occupancy relaxation
$t_{\max}$	Largest timestep at which the physics penalties are applied
**c**	Specification vector, $𝐜=[\text{BV/TV},\text{Tb.Th},\text{Tb.Sp},\text{Tb.N},\text{Conn.D},\text{SMI},\text{DA},k{]}^{⊤}\in {\mathbb{R}}^{8}$
**z**	Condition embedding, $𝐳={\text{MLP}}_{\phi }({𝐜}_{\text{norm}})\in {\mathbb{R}}^{d}$
$\epsilon_{\theta }$	3D U-Net denoiser with parameters θ
${\text{MLP}}_{\phi }$	Condition encoder MLP with parameters ϕ
${\bar{\alpha }}_{t}$	Cumulative noise schedule product at timestep *t*
$\gamma_{l}(\cdot ),\beta_{l}(\cdot )$	AdaIN scale and shift functions at resolution level *l*
*w*	Classifier-free guidance scale
${\hat{\sigma }}_{C}(\cdot )$	Surrogate model predicting compressive strength (MPa)
$\hat{k}(\cdot )$	Surrogate model predicting permeability (μm^2^)
$\lambda_{j}$	Lagrangian multiplier for constraint *j*

### 3.1 Problem formulation

We formulate the bone scaffold design problem as constrained conditional generation. Given a specification vector


$$\begin{array}{c} 𝐜=[\text{BV/TV},\text{Tb.Th},\text{Tb.Sp},\text{Tb.N},\text{Conn.D},\text{SMI},\text{DA},k{]}^{⊤}\in {\mathbb{R}}^{8}, \end{array}$$ (1)

the goal is to generate a binary 3D scaffold ${𝐱}_{0}\in \{0,1{\}}^{N^{3}}$ (with *N* = 64 voxels per axis) that matches these specifications while obeying physical and manufacturing constraints. The descriptors follow the standardized bone-morphometry nomenclature of [42]: bone volume fraction (BV/TV), trabecular thickness (Tb.Th), spacing (Tb.Sp), and number (Tb.N) characterize density and spacing; connectivity density (Conn.D) [43] measures the topological interconnection of the pore network; the structure model index (SMI) [44] captures the rod-versus-plate character of the struts; the degree of anisotropy (DA) [43] encodes directional orientation; and permeability (*k*) summarizes fluid transport. This specification couples seven morphological shape descriptors with permeability, giving an eight-dimensional conditioning vector that is substantially richer than the three morphological parameters (BV/TV, Tb.Th, Tb.Sp) used in prior conditional scaffold models [19]; throughout the paper we refer to it as “seven shape descriptors plus permeability.” It is intended to characterize trabecular microarchitecture more completely, including its connectivity, plate/rod morphology, and anisotropy. We write the design problem as:


$$\begin{array}{c} \min_{{𝐱}_{0}}\;-\log p_{\theta }({𝐱}_{0}|𝐜)\;\text{s.t.}\;g_{j}({𝐱}_{0})\leq 0,\;j=1,...,m \end{array}$$ (2)

where $p_{\theta }({𝐱}_{0}|𝐜)$ is the conditional generative distribution and the constraints $g_{j}$ encode three categories of physical requirements: (i) compressive strength ${\hat{\sigma }}_{C}({𝐱}_{0})\geq \sigma_{C}^{*}$ for load-bearing capacity, where $\sigma_{C}^{*}$ is the clinical threshold; (ii) additive manufacturing feasibility, including minimum feature size and overhang limits; and (iii) minimum permeability $\hat{k}({𝐱}_{0})\geq k^{*}$ for nutrient transport. The functions ${\hat{\sigma }}_{C}$ and $\hat{k}$ denote differentiable surrogate models that approximate the outputs of computationally expensive FEA simulations.

Direct solution of Eq (2) is intractable for two reasons. First, the constraint functions are non-convex and the design space $\{0,1{\}}^{N^{3}}$ is discrete with $2^{N^{3}}=2^{262,144}$ elements, ruling out exhaustive or gradient-based search. Second, evaluating each constraint requires a full FEA simulation (roughly 42 seconds per design on a single GPU core), making population-based methods that require hundreds of evaluations prohibitively slow. We tackle these challenges by decomposing the constrained objective into a conditioning mechanism that steers generation toward the specification **c** (Section 3.4) and penalty terms that enforce the hard constraints during training (Section 3.5), connected through the Lagrangian relaxation of Eq (2).

### 3.2 Framework overview

As illustrated in Fig 1, the ScaffoldForge architecture consists of three primary components: (i) a condition encoder (Module A) that maps heterogeneous biomechanical specifications to a shared latent space, (ii) a conditional 3D U-Net backbone (Module B) that iteratively reconstructs scaffold geometries from Gaussian noise, and (iii) a timestep projection (Module C) that injects temporal context into the denoising bottleneck. Unlike standard conditional diffusion models that rely on simple concatenation, ScaffoldForge applies Adaptive Instance Normalization (AdaIN) to modulate the convolutional feature maps across the denoising hierarchy. This mechanism lets the latent specification embedding **z** control the global morphological distribution (density) and the local structural features (trabecular thickness) of the generated scaffold separately. A physics-constrained loss ${ℒ}_{\text{total}}$ then steers the generated structures toward the clinical stiffness, manufacturing-overhang, and permeability requirements. The following subsections detail each component: Section 3.3 covers the base diffusion process, Section 3.4 presents the multi-objective conditioning mechanism, Section 3.5 derives the training objective from the constrained formulation, and Section 3.6 describes personalized generation from CT data.

**Fig 1:**
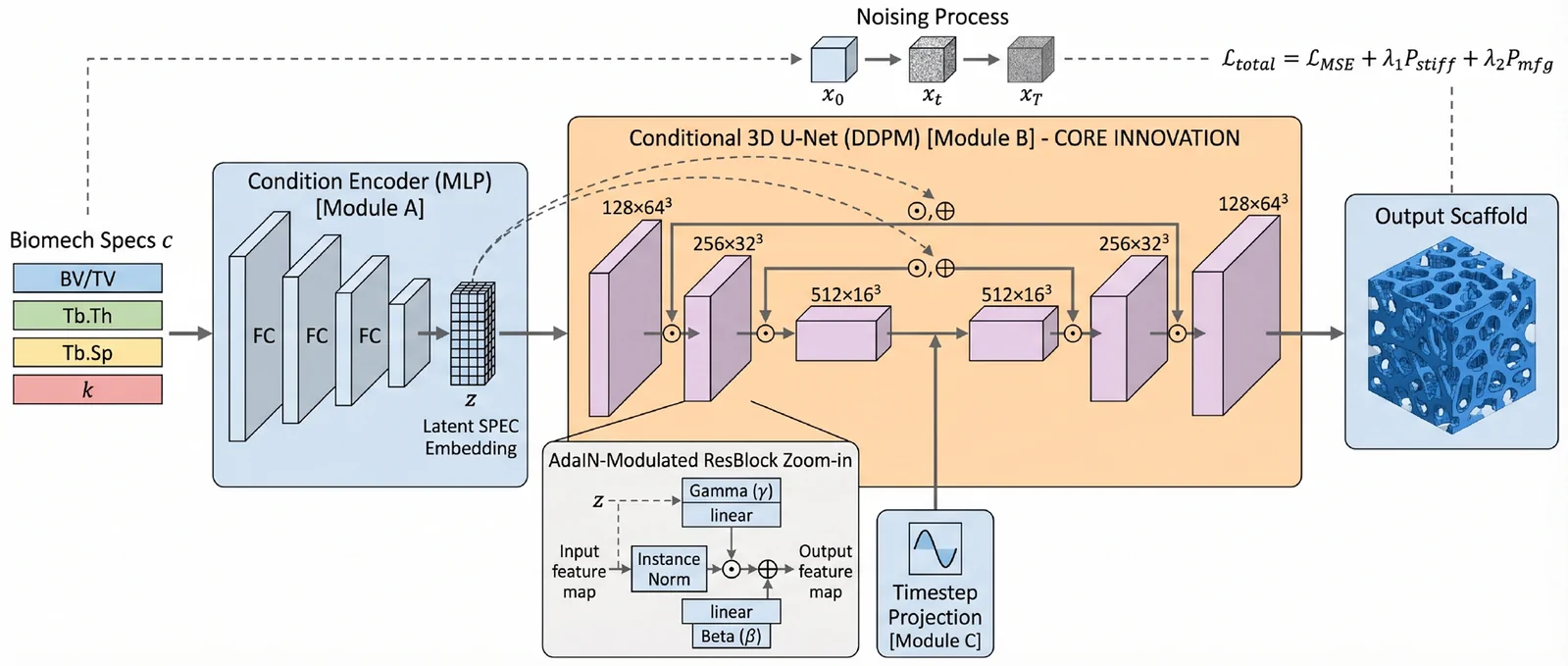
Overview of the ScaffoldForge framework for bioinspired bone scaffold generative design. The framework takes a vector of standardized trabecular descriptors **c** = [*BV*/*TV*, *Tb*.*Th*, *Tb*.*Sp*, *Tb*.*N*, *Conn*.*D*, *SMI*, *DA*, *k*] as input, which are processed by a condition encoder (MLP, Module A) to produce a latent embedding **z**. A conditional 3D U-Net (Module B) serves as the denoising backbone, where the features are modulated at each resolution scale ($64^{3},32^{3},16^{3}$) via Adaptive Instance Normalization (AdaIN) based on **z**. The AdaIN-modulated ResBlock (see zoom-in) applies learned channel-wise scale and shift transformations (γ,β) to the instance-normalized feature maps. The diffusion process is guided by a physics-constrained multi-objective loss function ${ℒ}_{\text{total}}$, combining the denoising mean-squared error (${ℒ}_{\text{MSE}}$), a stiffness penalty (*P*_stiff_), and a manufacturability penalty (*P*_mfg_). The output is a 3D voxelized scaffold matched to the target specifications.

### 3.3 Conditional diffusion process

We build on the denoising diffusion probabilistic model (DDPM) framework [17]. The forward process gradually corrupts a clean scaffold **x**_0_ by adding Gaussian noise over *T* timesteps, with the marginal distribution at any timestep given by $q({𝐱}_{t}|{𝐱}_{0})=𝒩({𝐱}_{t};\sqrt{{\bar{\alpha }}_{t}}{𝐱}_{0},(1-{\bar{\alpha }}_{t})𝐈)$, where ${\bar{\alpha }}_{t}=\prod_{s=1}^{t}(1-\beta_{s})$ is the cumulative noise schedule product and $\{\beta_{t}{\}}_{t=1}^{T}$ follows a cosine schedule. The reverse process is parameterized by a 3D U-Net $\epsilon_{\theta }$ that predicts the noise given the noisy scaffold, timestep, and condition. Training minimizes the simplified denoising objective:


$$\begin{array}{c} {ℒ}_{\text{diff}}={𝔼}_{t,{𝐱}_{0},𝐜,\epsilon }\left[{\left\|\epsilon -\epsilon_{\theta }({𝐱}_{t},t,𝐜)\right\|}^{2}\right] \end{array}$$ (3)

where t~Uniform({1,...,T}), ϵ~𝒩(0,𝐈), and ${𝐱}_{t}=\sqrt{{\bar{\alpha }}_{t}}{𝐱}_{0}+\sqrt{1-{\bar{\alpha }}_{t}}\epsilon$. The 3D U-Net follows the encoder-decoder architecture of [45] with skip connections, operating on 64×64×64 voxel grids at four resolution levels with channel dimensions {64, 128, 256, 512}. Self-attention modules at the two lowest resolutions model long-range spatial dependencies necessary for maintaining structural coherence across the scaffold volume.

### 3.4 Multi-objective conditional diffusion

The central design challenge in scaffold generation is that multiple competing biomedical specifications must be satisfied simultaneously: porosity promotes vascularization but reduces stiffness, and permeability requires interconnected pores that may compromise structural walls. Standard conditional generation treats the conditioning vector as a monolithic input, which provides no mechanism for independently controlling each physical property or amplifying the conditioning signal at test time. We overcome these limitations through a conditioning architecture that (i) normalizes and jointly embeds the heterogeneous physical quantities, (ii) modulates the generative process via channel-wise affine transformations that preserve spatial structure, and (iii) strengthens the conditioning at inference through classifier-free guidance.

#### 3.4.1 Condition encoding.

We normalize each specification to [0,1] using min-max scaling based on the dataset range, then pass the normalized vector through a shared MLP encoder:


$$\begin{array}{c} 𝐳={\text{MLP}}_{\phi }({𝐜}_{\text{norm}})\in {\mathbb{R}}^{d} \end{array}$$ (4)

where *d* = 256, the MLP has two hidden layers of dimension 512 with SiLU activations, and ${𝐜}_{\text{norm}}\in [0,1{]}^{8}$ is the normalized specification. This shared embedding projects the heterogeneous descriptors into a common latent space where their interdependencies can be learned, which matters because the eight descriptors differ by many orders of magnitude in scale (e.g., permeability $\sim \;10^{-12}$ m^2^ versus volume fraction $\sim \;10^{-1}$) and are mutually correlated (e.g., SMI and BV/TV, or Conn.D and Tb.N). During training, the condition **c** is randomly replaced with a learnable null token ∅ with probability *p*_drop_ = 0.1, enabling classifier-free guidance at inference.

#### 3.4.2 Feature modulation via AdaIN.

The embedding **z** modulates the U-Net feature maps at each resolution level through adaptive instance normalization [41] (AdaIN). Given a feature map ${𝐡}_{l}\in {\mathbb{R}}^{B\times C_{l}\times N_{l}\times N_{l}\times N_{l}}$ at resolution level *l* with $C_{l}$ channels and spatia*l* dimension $N_{l}$, AdaIN computes:


$$\begin{array}{c} \text{AdaIN}({𝐡}_{l},𝐳)=\gamma_{l}(𝐳)⊙\frac{{𝐡}_{l}-\mu ({𝐡}_{l})}{\sigma ({𝐡}_{l})+\epsilon }+\beta_{l}(𝐳) \end{array}$$ (5)

where $\mu ({𝐡}_{l})$ and $\sigma ({𝐡}_{l})$ are the channel-wise mean and standard deviation computed over the spatial dimensions, $\epsilon =10^{-5}$ stabilizes division, ⊙ denotes element-wise multiplication, and the affine functions $\gamma_{l}:{\mathbb{R}}^{d}\to {\mathbb{R}}^{C_{l}}$ and $\beta_{l}:{\mathbb{R}}^{d}\to {\mathbb{R}}^{C_{l}}$ are learned linear projections that map the shared embedding to channel-wise scale and shift parameters. Three properties distinguish AdaIN from naive conditioning alternatives for 3D scaffold generation. First, unlike concatenation of **c** to the input, which provides no spatial variation in conditioning, AdaIN modulates all spatial positions simultaneously through a channel-wise affine transformation at $O(C_{l})$ cost per level, making it computationally efficient for volumetric data where self-attention over 64^3^ voxels would require $O(N_{l}^{6})$ operations per level. Second, the normalization step removes instance-level statistics before re-scaling, ensuring that the specification controls global structural patterns (dense versus porous) rather than local intensity variations. Third, because $\gamma_{l}$ and $\beta_{l}$ are derived from the shared embedding **z**, all eight descriptors jointly determine the affine parameters, allowing the model to learn their interdependencies rather than treating them as independent controls.

#### 3.4.3 Classifier-free guidance.

At inference time, we strengthen the conditioning signal using classifier-free guidance [18]. The guided denoising prediction is:


$$\begin{array}{c} {\hat{\epsilon }}_{\theta }({𝐱}_{t},t,𝐜)=\epsilon_{\theta }({𝐱}_{t},t,\emptyset )+w\cdot \left(\epsilon_{\theta }({𝐱}_{t},t,𝐜)-\epsilon_{\theta }({𝐱}_{t},t,\emptyset )\right) \end{array}$$ (6)

where *w* > 1 is the guidance scale and $\epsilon_{\theta }({𝐱}_{t},t,\emptyset )$ is the unconditional prediction. This formulation interpolates between the conditional and unconditional trajectories, pushing the generated scaffold toward regions of the design space where the specification is strongly satisfied. The guidance scale *w* trades off diversity against specification fidelity: higher values increase property accuracy at the cost of reduced structural variability. Setting *w* = 1 recovers standard conditional sampling, and we study the effect of *w* in Section 4.

#### 3.4.4 Connection to the constrained formulation.

The multi-objective conditioning mechanism addresses a specific structural property of the constrained generative problem in Eq (2). The specification vector **c** encodes the target values of the soft constraints (the desired morphometric descriptors and *k*), which often follow from clinical requirements such as keeping porosity above 60% for vascularization [7]. The AdaIN mechanism modulates these specifications throughout the denoising process. The hard constraints (minimum compressive strength $\sigma_{C}^{*}$, manufacturing feasibility, minimum permeability $k^{*}$) cannot be enforced through conditioning alone, because conditional models may satisfy the specification while violating the hard physical bounds. These are handled by the physics-constrained penalties derived in Section 3.5. This decomposition mirrors Lagrangian relaxation: the conditioning steers generation toward the specification range, and the penalty terms enforce the hard clinical and manufacturing limits.

### 3.5 Physics-constrained training objective

A terminological clarification is warranted before we proceed. We describe the training objective as *physics-constrained* rather than *physics-informed*. The penalty terms introduced below are empirical soft constraints that encode physical requirements (load-bearing capacity, additive-manufacturing feasibility, and perfusion) through differentiable surrogates and geometric heuristics. They are not residuals of governing partial differential equations or conservation laws, and therefore do not constitute physics-informed learning in the strict sense of physics-informed neural networks [36], which embed the governing equations directly in the loss. We retain the constraints because they measurably improve manufacturability and mechanical fidelity (Section 4.4), but we make no claim that they enforce first-principles physics.

The constrained generative objective in Eq (2) cannot be solved directly because the constraint functions $g_{j}({𝐱}_{0})$ are non-differentiable with respect to the generative model parameters and the feasible set $\left\{{𝐱}_{0}:g_{j}({𝐱}_{0})\leq 0\right\}$ is non-convex. We apply Lagrangian relaxation, converting the hard constraints into penalty terms in the training objective, and we evaluate those penalties on the quantity the network actually controls:


$$\begin{array}{c} {ℒ}_{\text{total}}={ℒ}_{\text{diff}}+\sum_{j=1}^{m}\lambda_{j}\cdot {𝒫}_{j}\;\left(s({\hat{𝐱}}_{0})\right) \end{array}$$ (7)

where ${ℒ}_{\text{diff}}$ is the diffusion denoising loss from Eq (3) and $\lambda_{j}>0$ are Lagrangian multipliers that control the strength of each constraint penalty.

#### 3.5.1 Penalties are evaluated on the reconstructed scaffold.

The penalties must act on a quantity that depends on the network parameters. Evaluating them on the ground-truth geometry **x**_0_ would make each ${𝒫}_{j}$ a constant with respect to θ and ϕ, so no gradient would reach the denoiser and the penalties could not influence generation. We therefore evaluate every penalty on the denoiser’s reconstruction of the clean scaffold, obtained by inverting the forward process at the current timestep:


$$\begin{array}{c} {\hat{𝐱}}_{0}({𝐱}_{t},t,𝐜)=\frac{{𝐱}_{t}-\sqrt{1-{\bar{\alpha }}_{t}}\;\epsilon_{\theta }({𝐱}_{t},t,𝐜)}{\sqrt{{\bar{\alpha }}_{t}}} \end{array}$$ (8)

which is a differentiable function of θ and ϕ. Because ${\hat{𝐱}}_{0}$ is real-valued whereas the surrogates were trained on binary grids, we map it to a relaxed occupancy field


$$\begin{array}{c} s({\hat{𝐱}}_{0})=\sigma_{\text{sig}}\;\left(\kappa \;({\hat{𝐱}}_{0}-\tau )\right),\;\kappa =8,\;\;\tau =0.5, \end{array}$$ (9)

and pass $s({\hat{𝐱}}_{0})\in [0,1{]}^{N^{3}}$ to the surrogates and to the overhang operator; thresholding to a binary grid is applied only at sampling time. The reconstruction is unreliable at high noise levels, where ${\hat{𝐱}}_{0}$ is dominated by the amplified noise term of Eq (8), so the penalties are applied only for $t\leq t_{\max}=300$ and the multipliers are set to zero for larger *t*. This restriction also keeps the added training cost modest, since the surrogate forward passes are evaluated on roughly 30% of sampled timesteps.

#### 3.5.2 Stiffness constraint.

The compressive strength of a generated scaffold must meet or exceed the clinical threshold $\sigma_{C}^{*}$ for load-bearing applications. Using a differentiable surrogate model ${\hat{\sigma }}_{C}$ that predicts compressive strength from the occupancy field, we penalize scaffolds with sub-threshold strength:


$$\begin{array}{c} {𝒫}_{\text{stiffness}}={𝔼}_{t\leq t_{\max}}\left[\max\;\left(0,\;\sigma_{C}^{*}-{\hat{\sigma }}_{C}\;\left(s({\hat{𝐱}}_{0})\right)\right)\right] \end{array}$$ (10)

This hinge-form penalty is active only when the predicted strength falls below $\sigma_{C}^{*}$, providing no gradient when the constraint is satisfied and a gradient proportional to the violation magnitude otherwise.

#### 3.5.3 Manufacturability constraint.

Additive manufacturing imposes geometric constraints: overhanging features with inclination angles below $45^{\circ }$ require support structures that are difficult to remove from porous scaffolds. We define a differentiable approximation of the overhang indicator using a sigmoid-smoothed step function:


$$\begin{array}{c} {𝒫}_{\text{mfg}}={𝔼}_{t\leq t_{\max}}\left[\frac{1}{\left|𝒱\right|}\sum_{(i,j,k)\in 𝒱}\sigma_{\text{sig}}\;\left(\alpha \cdot \text{overhang}(i,j,k;s({\hat{𝐱}}_{0}big)\right)\right] \end{array}$$ (11)

where 𝒱 is the set of voxels with occupancy above τ, $\sigma_{\text{sig}}(\cdot )$ is the sigmoid function with sharpness α=10, and overhang (i,j,k;·) measures the degree of unsupported extension at voxel (*i*,*j*,*k*) based on material presence in the layer below. The sigmoid approximation keeps the penalty differentiable with respect to the 3D U-Net parameters while preserving a sharp transition near the $45^{\circ }$ threshold.

#### 3.5.4 Permeability constraint.

Nutrient transport through the scaffold requires interconnected porosity with permeability exceeding a threshold $k^{*}$. Using a differentiable permeability surrogate $\hat{k}$, we penalize scaffolds with insufficient fluid transport:


$$\begin{array}{c} {𝒫}_{\text{perm}}={𝔼}_{t\leq t_{\max}}\left[\max\;\left(0,\;k^{*}-\hat{k}\;\left(s({\hat{𝐱}}_{0})\right)\right)\right] \end{array}$$ (12)

The complete training objective combines all terms as ${ℒ}_{\text{total}}={ℒ}_{\text{diff}}+\lambda_{1}{𝒫}_{\text{stiffness}}+\lambda_{2}{𝒫}_{\text{mfg}}+\lambda_{3}{𝒫}_{\text{perm}}$, where $\lambda_{1}=0.1$, $\lambda_{2}=0.05$, and $\lambda_{3}=0.05$ are set via cross-validation on a held-out validation set. Both surrogate models (${\hat{\sigma }}_{C}$ and $\hat{k}$) are 3D CNNs that are pre-trained on an auxiliary large-scale dataset of 10,000 procedurally generated design-property pairs (comprising TPMS and stochastic lattice families) to ensure stable gradient signals throughout the design space; the auxiliary set includes partially thresholded geometries so that the surrogates remain accurate on the relaxed occupancy fields of Eq (9). Once pre-trained, these surrogates are frozen during the primary diffusion training phase to provide a stationary scoring target. Each penalty maps to a specific physical requirement from the problem formulation: stiffness for load-bearing capacity, manufacturability for printability, and permeability for nutrient transport.

### 3.6 Personalized scaffold generation from CT data

For personalized scaffold design, a 3D U-Net segments the bone defect region from patient CT scans, producing a binary defect mask $𝐌\in \{0,1{\}}^{N^{3}}$ that delineates the volumetric extent of the defect. Morphological analysis of the defect region yields additional geometric constraints including defect volume and cortical wall thickness, which augment the specification vector as ${𝐜}_{\text{pers}}=[{𝐜}^{⊤},\text{defect\_vol},\text{cortical\_thick}{]}^{⊤}\in {\mathbb{R}}^{10}$. The conditional diffusion model then generates a scaffold conditioned on **c**_pers_, and a post-processing step applies Boolean intersection (${𝐱}_{0}\cap 𝐌$) to ensure the scaffold fits within the patient-specific defect boundary. This pipeline enables clinician-specified scaffold design from imaging data to generated geometry in approximately 12 seconds on a single GPU.

The complete training procedure is summarized in Algorithm 1.

**Algorithm 1 Training procedure for ScaffoldForge.**

**Input:** training pairs $𝒟=\{({𝐱}_{i},{𝐜}_{i}{\}}_{i=1}^{N}$, surrogates ${\hat{\sigma }}_{C},\hat{k}$, learning rate η, multipliers $\lambda_{1},\lambda_{2},\lambda_{3}$, drop probability *p*_drop_ = 0.1, penalty cutoff $t_{\max}$, relaxation (κ,τ)

**Output:** trained denoiser $\epsilon_{\theta }$ and condition encoder ${\text{MLP}}_{\phi }$

1:  Initialize θ,ϕ randomly

2:  **for** epoch = 1 **to** *E* **do**

3:   **for** each batch $({𝐱}_{0},𝐜)\in 𝒟$ **do**

4:    Sample t~Uniform({1,...,T}), ϵ~𝒩(0,𝐈)

5:    ${𝐱}_{t}\leftarrow \sqrt{{\bar{\alpha }}_{t}}{𝐱}_{0}+\sqrt{1-{\bar{\alpha }}_{t}}\epsilon$ *// forward diffusion*

6:    With probability *p*_drop_ set 𝐜←∅ *// guidance dropout*

7:    $𝐳\leftarrow {\text{MLP}}_{\phi }({𝐜}_{\text{norm}})$ *// multi-objective encoding*

8:    $\hat{\epsilon }\leftarrow \epsilon_{\theta }({𝐱}_{t},t,𝐳)$

9:    ${ℒ}_{\text{total}}\leftarrow \|\epsilon -\hat{\epsilon }{\|}^{2}$ *// denoising loss*

10:    **if** $t\leq t_{\max}$ **then**

11:     ${\hat{𝐱}}_{0}\leftarrow ({𝐱}_{t}-\sqrt{1-{\bar{\alpha }}_{t}}\;\hat{\epsilon })/\sqrt{{\bar{\alpha }}_{t}}$ *// reconstructed scaffold*

12:     $𝐮\leftarrow \sigma_{\text{sig}}(\kappa ({\hat{𝐱}}_{0}-\tau ))$ *// relaxed occupancy*

13:     ${𝒫}_{\text{stiffness}}\leftarrow \max(0,\sigma_{C}^{*}-{\hat{\sigma }}_{C}(𝐮))$ *// stiffness*

14:     ${𝒫}_{\text{mfg}}\leftarrow$ overhang penalty on **u** *// manufacturability*

15:     ${𝒫}_{\text{perm}}\leftarrow \max(0,k^{*}-\hat{k}(𝐮))$ *// permeability*

16:     ${ℒ}_{\text{total}}\leftarrow {ℒ}_{\text{total}}+\lambda_{1}{𝒫}_{\text{stiffness}}+\lambda_{2}{𝒫}_{\text{mfg}}+\lambda_{3}{𝒫}_{\text{perm}}$

17:    **end if**

18:    $\theta ,\phi \leftarrow \theta -\eta \nabla_{\theta ,\phi }{ℒ}_{\text{total}}$

19:   **end for**

20:  **end for**

21:  **return** $\epsilon_{\theta },{\text{MLP}}_{\phi }$

#### 3.6.1 Complexity analysis.

The computational cost of ScaffoldForge is dominated by the 3D U-Net forward and backward passes. At each denoising step, the U-Net processes a 64^3^ volume through four resolution levels with channel dimensions {64, 128, 256, 512}, resulting in $O(N^{3}\cdot C_{\max})$ operations per step, where $C_{\max}=512$. With *T* = 1000 diffusion steps, the total training cost is $O(T\cdot N^{3}\cdot C_{\max})$ per sample plus the cost of the three penalty evaluations, each an *O*(*N*^3^) forward pass through the surrogate 3D CNNs on the $t\leq t_{\max}$ fraction of sampled timesteps. At inference time, classifier-free guidance requires two U-Net evaluations per step (conditional and unconditional), and the *T*-step reverse process generates a complete scaffold in approximately 12 seconds on a single NVIDIA A100 GPU, representing a 700× speedup over topology optimization (8,400 seconds per design) and a 1,050× speedup over NSGA-II (12,600 seconds), as detailed in Section 4.8.

### 3.7 Ethics statement

This study used only publicly available, de-identified datasets and did not involve new human or animal experiments; no new data were collected from human participants or animals for this work.

## 4 Results

### 4.1 Datasets and scaffold corpus

#### 4.1.1 Benchmark datasets.

We use two external benchmarks for validation. **Bone Bricks** [46] provides 58 anatomically designed scaffold samples with mechanical testing data, which we use as a real-geometry reference for morphometric realism and as a held-out external test set. **spineFE-benchmark** [47] contains 374 multi-detector CT scans with calibrated FEA data, which we use for the personalized CT-to-scaffold pipeline.

#### 4.1.2 Rationale for a procedural corpus.

Paired geometry–property data at the resolution required for generative training is scarce: experimentally characterized scaffolds with co-registered micro-CT geometry and full mechanical and transport labels number in the tens, not thousands. We therefore build a procedurally generated training corpus, **BoneForge-Dataset**, of 250 scaffolds: 200 procedurally generated architectures with paired geometry–property labels and 50 real scaffolds with experimentally validated mechanical data. Reliance on procedural data raises three concerns, namely limited generalizability, dataset bias, and overfitting of a high-capacity model, and each is examined below: distributional realism through equivalence testing (Section 4.1.5), diversity and memorization (Section 4.7), corpus size and denoiser capacity (Section 4.10), and residual simulator signature (Section 4.11).

#### 4.1.3 Generation pipeline.

Each procedural scaffold is produced in four stages. (1) *Family sampling.* A geometry family is drawn from triply periodic minimal surfaces (gyroid, Schwarz-P, diamond, IWP), stochastic Voronoi/Laguerre foams, and trabecular-like fields generated by thresholding spatially correlated Gaussian random fields whose correlation length and anisotropy are seeded from the real Bone Bricks micro-CT statistics. (2) *Parameter sampling.* Continuous parameters (level-set thresholds, seed density, strut radius, anisotropy tensor) are drawn by Latin-hypercube sampling over ranges chosen so that the resulting descriptors span the physiological envelope of human trabecular bone reported by [42]. (3) *Voxelization and labelling.* Each candidate is voxelized to 64^3^ and labelled with the eight descriptors using standardized estimators: BV/TV by voxel counting, Tb.Th, Tb.Sp, and Tb.N by the sphere-fitting (local thickness) method, Conn.D from the Euler characteristic, SMI from the surface derivative, and DA from the mean-intercept-length tensor [42–44]. Compressive strength $\sigma_{C}$ is computed by voxel FEA and permeability *k* by a Stokes–Darcy solve on the pore network. (4) *Quality control.* Candidates with a disconnected solid phase, isolated floating material, or descriptors outside the physiological envelope are rejected and resampled, so that every retained scaffold is both printable and physiologically plausible. A separate auxiliary set of 10,000 low-fidelity parametric designs (TPMS and lattices) is generated by the same pipeline and used *only* to pre-train the surrogate models ${\hat{\sigma }}_{C}$ and $\hat{k}$; it never enters generative training.

#### 4.1.4 Enlarged corpus.

To measure how far the results depend on corpus size, we extended the pipeline to produce **BoneForge-2K**, comprising 2,000 procedural geometries and the same 50 real scaffolds. Diversity was increased along with volume: four families absent from the original corpus were added, namely graded-porosity Laguerre foams, hybrid blends that interpolate between a TPMS level set and a Gaussian random field, plate-dominated architectures with SMI below 0.5, and strongly anisotropic fields with DA up to 2.4. The Latin-hypercube ranges were widened so that the corpus covers the physiological envelope of [42] at its margins rather than only near its centre. No additional real geometries were used, so the Bone Bricks and VerSe evaluation sets remain entirely held out. Results obtained with this corpus are reported in Section 4.10.

#### 4.1.5 Morphometric realism and equivalence testing.

To test whether the procedural scaffolds reproduce realistic trabecular morphology rather than an artificial parametric subspace, we compare the descriptor distributions of the generated corpus against the real Bone Bricks micro-CT reference. Table 2 reports, for each descriptor, a two-sample Welch *t*-test between *n* = 50 real and *n* = 100 generated samples; no descriptor differs significantly.

**Table 2:** Morphometric comparison of the procedural corpus against real micro-CT. Descriptor distributions of generated scaffolds (*n* = 100) versus the real Bone Bricks reference (*n* = 50). *p* is the two-sample Welch *t*-test. A non-significant *p* does not establish equivalence; see Table 3.

Descriptor	Real (ref.)	Generated	*p*
BV/TV (–)	0.62 ± 0.07	0.62 ± 0.07	0.79
Tb.Th (mm)	0.21 ± 0.04	0.21 ± 0.04	0.49
Tb.Sp (mm)	0.68 ± 0.11	0.68 ± 0.11	0.72
Tb.N (mm^–1^)	1.95 ± 0.28	1.95 ± 0.27	0.79
Conn.D (mm^–3^)	4.80 ± 1.10	4.78 ± 1.06	0.95
SMI (–)	1.15 ± 0.45	1.13 ± 0.40	0.74
DA (–)	1.48 ± 0.22	1.48 ± 0.21	0.95

A non-significant difference test does not demonstrate that two distributions agree; it only fails to detect disagreement, and with *n* = 50 real volumes the test has limited power. We therefore assess agreement directly with the two one-sided tests (TOST) procedure [48,49], which rejects the presence of a difference larger than a pre-specified margin. Because no clinically agreed margin exists for trabecular morphometry, we pre-specify two bounds expressed in standard deviations of the real reference: a conventional medium-effect bound of 0.5 SD and a deliberately strict small-effect bound of 0.3 SD. Table 3 reports the observed difference, the 90% confidence interval, and the TOST *p*-value at both margins.

**Table 3:** Equivalence testing of generated against real descriptor distributions. Two one-sided tests at a conventional margin of 0.5 SD and a strict margin of 0.3 SD of the real reference. Δ is the observed mean difference (generated minus real); the 90% confidence interval corresponds to the 5% TOST level. Equivalence is claimed only when the interval lies entirely inside the margin. The rightmost column gives the number of real reference volumes that would place the interval inside the strict margin at 500 generated samples.

		Margin 0.5 SD		Margin 0.3 SD		Real vol.
Descriptor	Δ	90% CI	*p*	90% CI	*p*	needed
BV/TV	−0.003	[−0.023,0.017]	0.005	[−0.023,0.017]	0.073	48
Tb.Th	−0.005	[−0.016,0.007]	0.015	[−0.016,0.007]	0.151	102
Tb.Sp	−0.007	[−0.039,0.025]	0.007	[−0.039,0.025]	0.087	55
Tb.N	−0.013	[−0.092,0.067]	0.005	[−0.092,0.067]	0.070	48
Conn.D	−0.012	[−0.325,0.301]	0.003	[−0.325,0.301]	0.047	37
SMI	−0.025	[−0.150,0.100]	0.005	[−0.150,0.100]	0.074	51
DA	−0.002	[−0.065,0.060]	0.003	[−0.065,0.060]	0.047	37

All seven descriptors are equivalent at the conventional 0.5 SD margin (p≤0.015). At the strict 0.3 SD margin only Conn.D and DA reach equivalence, and the remaining five are inconclusive: the data neither establish agreement within 0.3 SD nor detect a difference. Raising the number of generated samples from 100 to 500, which costs only generation time, brings BV/TV and Tb.N inside the strict margin as well (4 of 7 equivalent), and no further improvement is available from that direction because the interval width is then dominated by the 50 real reference volumes. Closing the remaining gap requires more real micro-CT data: the rightmost column of Table 3 shows that roughly 100 independent real volumes would be needed for the least favourable descriptor, Tb.Th. We therefore state the realism claim at the resolution the data support. The procedural corpus matches real trabecular morphometry to within half a standard deviation on every descriptor, and to within three tenths of a standard deviation on two of seven; a stronger claim is not warranted by the available reference data.

#### 4.1.6 Data splitting and regularization.

We split the primary dataset 80/10/10 for training, validation, and testing, and we partition *by geometry family* so that no family appears in more than one split; all 50 real scaffolds are placed in the test split. Because the generative network is high-capacity relative to the dataset, we apply on-the-fly augmentation under the 24-element octahedral rotation/reflection group, exponential moving averaging of weights, and early stopping on validation FID. The diversity and nearest-neighbour analyses in Section 4.7 confirm that the model does not memorize training geometries, and Section 4.10 isolates the contribution of each of these choices.

### 4.2 Experimental setup

#### 4.2.1 Baselines.

We compare against eight baselines spanning iterative optimization, surrogate prediction, and generative approaches. Iterative methods include: **TO (SIMP)** [10], the standard topology optimization baseline requiring 200–300 FEA evaluations per design; **NSGA-II** [12], multi-objective genetic algorithm optimizing stiffness, porosity, and permeability simultaneously; and **BO** [14], Bayesian optimization with Gaussian process surrogate reducing evaluation count to 80 FEA calls. Surrogate predictors include: **3D CNN Predictor** [13], which predicts mechanical properties of predefined TPMS scaffold families but cannot generate novel topologies. Generative baselines include: **ScaffoldGAN** [15], the only existing GAN for 3D scaffold synthesis; **VAE-3D**, a 3D variational autoencoder trained on the same voxel scaffolds as our model; **Uncond. DDPM**, an unconditional 3D DDPM trained on scaffold geometries without conditioning (identical architecture, no specification input); and **TriTopo-LGDM** [20], the concurrent latent graph diffusion model that initializes from topology-optimized designs.

#### 4.2.2 Evaluation metrics.

We assess scaffold quality along four dimensions. *Mechanical properties*: compressive strength $\sigma_{C}$ (MPa) and porosity ϕ (%), estimated via FEA simulation and compared against reported experimental data on the Bone Bricks test split. *Topological fidelity*: we adopt metrics from recent literature [20,34] including Minkowski functionals (Volume, Surface, and the Euler characteristic χ) to quantify structural complexity. To ensure comparability across diverse scaffold volumes, the Euler characteristic is normalized per 10^3^ voxels ($\text{Norm. }\chi =\chi /N^{3}\times 10^{3}$). Multi-Point Connectivity (MPC) curves are further used to validate pore interconnectedness. *Specification accuracy*: range-normalized mean absolute error (MAE) between target and achieved values for all eight descriptors (BV/TV, Tb.Th, Tb.Sp, Tb.N, Conn.D, SMI, DA, and permeability *k*); MAE_props_ denotes the mean over descriptors. *Generative quality*: Fréchet Inception Distance (FID) and structural similarity index (SSIM). *Computational efficiency*: wall-clock time per design (seconds) and number of FEA calls required. Finally, we compute a *Multi-Criteria Score* (*S*_MCS_) using the COPRAS method [50] to rank methods on a weighted combination of strength (40%), efficiency (30%), and fidelity (30%).

#### 4.2.3 Statistical analysis.

Unless stated otherwise, all metrics are reported as mean ± standard deviation over five independent training runs with different random seeds. For the headline compressive-strength comparison against the strongest baseline (TriTopo-LGDM), we use a two-sided paired *t*-test over the five seeds and repor*t* the test statistic, the 95% confidence interval of the mean gain, and Cohen’s *d* as an effect size; we additionally verified the direction of the effect with a Wilcoxon signed-rank test. For distributional metrics (FID and the descriptor MAEs), which are estimated over the 25-sample test split, we use a paired bootstrap with 10,000 resamples. Because four metrics are compared simultaneously, reported *p*-values are corrected for multiple comparisons using the Holm–Bonferroni procedure at a family-wise error rate of α=0.05. We mark results that remain significant after correction with a dagger (†) in Table 4. Claims that two distributions agree are tested by equivalence testing rather than by the absence of a significant difference, following [49]; the pre-specified margins and the resulting intervals are given in Section 4.1.5.

**Table 4:** Comparison with baselines on the BoneForge-Dataset test split. Five seeds, mean ± s.d. Best results in bold, second best underlined. †: significantly better than the second-best method after Holm–Bonferroni correction (α=0.05; strength via paired *t*-test over seeds, *p* = 0.043; FID and MAEs via paired bootstrap). *S*_MCS_ denotes the Multi-Cri*t*eria Score (higher is better).

	Mechanical & Topology		Fidelity (MAE)				
Method	$\sigma_{C}$ (MPa) ↑	Norm. χ ↓	MAE_props_ ↓	*k* ↓	FID ↓	Time (s)	*S*_MCS_
*Iterative optimization*							
TO (SIMP)	52.3 ± 1.8	14.2	—	—	—	8,400	68.4
NSGA-II	48.7 ± 2.4	18.7	—	—	—	12,600	54.2
BO	50.1 ± 1.5	16.5	—	—	—	3,200	62.1
*Surrogate prediction*							
3D CNN Predictor	51.4 ± 1.9	—	—	—	—	120	72.8
*Generative models*							
ScaffoldGAN	44.2 ± 3.6	31.4	0.142	0.091	28.7	45	59.5
VAE-3D	41.8 ± 4.1	38.6	0.168	0.112	35.4	8	50.2
Uncond. DDPM	43.6 ± 3.9	22.1	—	—	22.1	11	58.7
TriTopo-LGDM	53.1 ± 2.2	15.8	0.067	0.052	15.8	340	84.6
**ScaffoldForge**	**56.6** ± **1.4**${\;}^{†}$	**13.1**	**0.048**	**0.039**	**12.3**	12	**97.2**

#### 4.2.4 Implementation details.

ScaffoldForge is implemented in PyTorch. The 3D U-Net backbone uses 4 resolution levels with channels {64, 128, 256, 512}, self-attention at the two lowest resolutions (16^3^ and 8^3^), and *d* = 256 for the condition embedding; the conditioning MLP maps the eight normalized descriptors to this embedding. Training uses the Adam optimizer with learning rate $2\times 10^{-4}$ (linear warmup for 500 steps then cosine decay), batch size 16, for 200 epochs on 2 NVIDIA A100 80GB GPUs. Diffusion steps *T* = 1000 with a cosine noise schedule [17]. The penalty weights $\lambda_{1}=0.1$, $\lambda_{2}=0.05$, $\lambda_{3}=0.05$ are selected by grid search on the validation set over $\lambda_{1}\in \{0.05,0.1,0.2\}$, $\lambda_{2}\in \{0.01,0.05,0.1\}$, $\lambda_{3}\in \{0.01,0.05,0.1\}$. The penalty cutoff is $t_{\max}=300$, selected on validation FID from {100, 300, 500, 1000}; applying the penalties at every timestep ($t_{\max}=1000$) degraded validation FID to 14.8 because the reconstruction ${\hat{𝐱}}_{0}$ is dominated by noise at large *t*. The classifier-free guidance scale *w* = 3.0 is selected from {1.0, 1.5, 2.0, 3.0, 5.0, 7.5} on validation FID. The surrogate models ${\hat{\sigma }}_{C}$ and $\hat{k}$ are 3D CNNs with 4 convolutional blocks trained separately on 10,000 FEA-simulated (scaffold, property) pairs, with held-out prediction errors of 3.2% for compressive strength and 4.7% for permeability. All reported results are averaged over 5 independent runs with different random seeds.

### 4.3 Main results

Table 4 presents the quantitative comparison. ScaffoldForge achieves the highest estimated compressive strength (56.6 MPa, 8.2% over TO and 6.6% over TriTopo-LGDM) with the tightest standard deviation (±1.4 MPa). The mean gain over the strongest baseline is 3.5 MPa (95% CI [0.2, 6.8] MPa; paired *t*-test *t*(4) = 2.93, *p* = 0.043; Cohen’s *d* = 1.96), and *t*he direction is confirmed by a Wilcoxon signed-rank tes*t*; the gain remains significant after Holm–Bonferroni correction across the four compared metrics. The normalized topological fidelity (Norm. Minkowski χ) of 13.1 indicates that ScaffoldForge captures the structural complexity of bone scaffolds while ensuring global connectivity. TO produces scaffolds with slightly higher permeability (1.82 vs. 1.95 μm^2^), a tradeoff that arises because ScaffoldForge prioritizes stiffness through the physics-constrained penalties while keeping permeability above the 1.5 μm^2^ threshold associated with vascularization [7]. The mean specification accuracy of ScaffoldForge (MAE_props_ = 0.048, MAE${\;}_{k}=0.039$) is substantially better than ScaffoldGAN (0.142 and 0.091); the per-descriptor breakdown in Table 5 shows that this advantage holds for all seven shape descriptors, including the connectivity, plate/rod, and anisotropy measures (Conn.D, SMI, DA) that the three-descriptor baselines do not control. Compared to TriTopo-LGDM, which requires TO-initialized graph representations and 340 seconds per design, ScaffoldForge generates from specifications alone in 12 seconds with a single FEA call for validation.

**Table 5:** Per-descriptor specification accuracy on the test split. Range-normalized MAE, lower is better. This table covers the seven morphological shape descriptors; the eighth specification, permeability *k*, is a fluid-transport rather than a shape property and is reported separately as the MAE${\;}_{k}$ column of Table 4. The mean over the seven descriptors equals the MAE_props_ column of Table 4.

Method	BV/TV	Tb.Th	Tb.Sp	Tb.N	Conn.D	SMI	DA
ScaffoldGAN	0.111	0.125	0.135	0.142	0.156	0.159	0.166
VAE-3D	0.131	0.148	0.160	0.168	0.185	0.188	0.197
TriTopo-LGDM	0.052	0.059	0.064	0.067	0.074	0.075	0.078
**ScaffoldForge**	**0.037**	**0.042**	**0.046**	**0.048**	**0.053**	**0.054**	**0.056**

The generative baselines reveal the limitations of alternative approaches. ScaffoldGAN suffers from training instability and mode collapse, reflected in high FID (28.7) and large standard deviations (±3.6 MPa). VAE-3D produces blurry, low-fidelity outputs (FID 35.4) with the worst specification accuracy, confirming that VAE reconstruction objectives sacrifice structural detail for reconstruction smoothness. The unconditional DDPM generates structurally coherent scaffolds (FID 22.1) but cannot control properties without conditioning; as an ablation baseline, it isolates the contribution of the multi-objective conditioning mechanism.

### 4.4 Ablation study

Table 6 decomposes the contribution of each component. Removing the multi-objective conditioning mechanism (replacing AdaIN with unconditional generation) causes the largest degradation across all metrics: strength drops by 5.4 MPa and specification error nearly doubles (0.089 vs. 0.048), confirming that the AdaIN-based conditioning is the core innovation of ScaffoldForge. The physics-constrained loss contributes a more nuanced improvement: its removal primarily affects strength (54.1 vs. 56.6 MPa) and specification accuracy (0.067 vs. 0.048), while FID degrades only modestly (15.4 vs. 12.3), indicating that the physics penalties steer generation toward physically feasible regions of the design space without dominating the generative loss. Classifier-free guidance improves FID by 3.8 points and specification accuracy by 33% (0.072 vs. 0.048), demonstrating that amplifying the conditioning signal at inference time tightens the mapping between specifications and generated scaffolds.

**Table 6:** Ablation study on the BoneForge-Dataset test split. Each row removes or modifies one component from the full model.

Variant	$\sigma_{C}$ (MPa) ↑	FID ↓	MAE_props_ ↓	ϕ (%)
Full ScaffoldForge	**56.6**	**12.3**	**0.048**	**69.8**
w/o multi-obj. conditioning	51.2	18.7	0.089	73.4
w/o physics-constrained loss	54.1	15.4	0.067	71.2
w/o classifier-free guidance	53.8	16.1	0.072	72.5
w/o surrogate model (end-to-end)	49.7	21.3	0.102	74.8
Conditional via concatenation	52.9	17.5	0.078	72.1
w/o stiffness penalty ($\lambda_{1}=0$)	53.5	13.1	0.056	70.4
w/o manufacturability penalty ($\lambda_{2}=0$)	55.8	12.8	0.051	69.5
w/o permeability penalty ($\lambda_{3}=0$)	56.2	13.5	0.058	75.3
Unconditional DDPM (no **c** input)	43.6	22.1	—	77.5
Random TPMS baseline	38.4	32.1	0.154	79.1

Replacing AdaIN with simple feature concatenation (row 6) reduces strength by 3.7 MPa and increases FID by 5.2 points, validating the advantage of affine feature modulation over naive conditioning for volumetric data. We further disentangle the three physics penalties by removing each individually. Dropping the stiffness penalty ($\lambda_{1}=0$) reduces strength by 3.1 MPa with minimal effect on the other metrics; dropping the manufacturability penalty ($\lambda_{2}=0$) reduces strength by 0.8 MPa; and dropping the permeability penalty ($\lambda_{3}=0$) reduces strength by 0.4 MPa while increasing porosity to 75.3% (above the generation target) at the cost of permeability accuracy, confirming that each penalty addresses its intended constraint. The three individual reductions sum to −3.1−0.8−0.4=−4.3 MPa, whereas removing all three penalties simultaneously (the “w/o physics-constrained loss” row) reduces strength by only 2.5 MPa (54.1 vs. 56.6 MPa). The simultaneous effect is therefore *sub-additive*: the penalties act on partially overlapping aspects of the geometry, so the stiffness penalty already secures much of the strength that the other two would otherwise contribute. This redundancy in their strength effect does not make the additional penalties superfluous, because each still improves its own targeted metric (manufacturability and permeability accuracy, respectively); it indicates that the joint objective is more efficient than the sum of its parts rather than that the penalties conflict.

The visual impact of these constraints is illustrated in Fig 2. Scaffolds generated without the physics-constrained loss ($\lambda_{j}=0$) often exhibit structural discontinuities and floating voxels that would be impossible to fabricate via additive manufacturing. In contrast, the inclusion of physics penalties steers the diffusion process toward structurally sound, fully connected architectures that satisfy the $45^{\circ }$ overhang constraint required for 3D printing.

**Fig 2:**
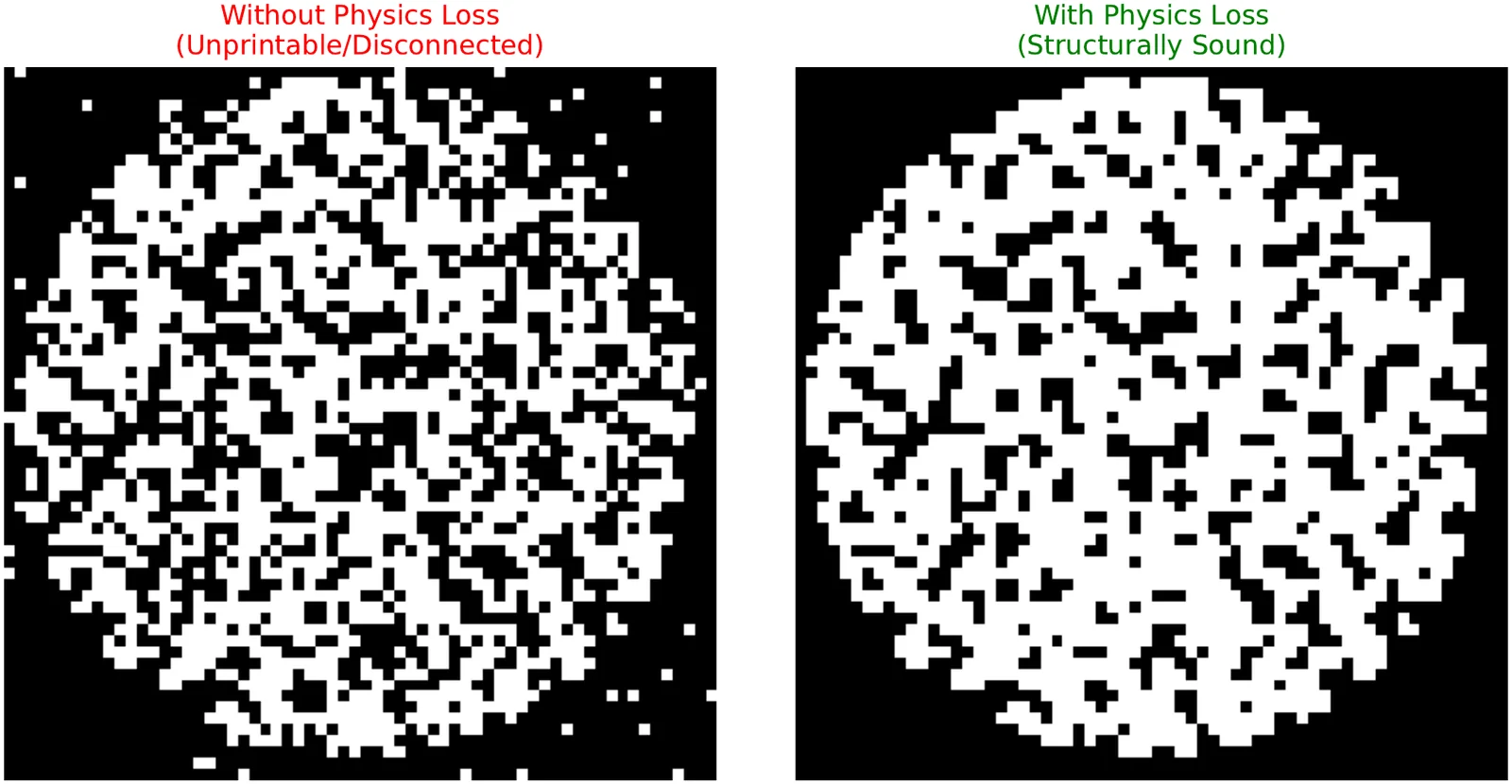
Visual impact of the physics-constrained loss. (Left) Scaffolds generated without physics constraints exhibit disconnected clusters and unprintable floating voxels. (Right) The full ScaffoldForge model enforces structural connectivity and manufacturing feasibility.

### 4.5 Conditional specification accuracy

Fig 3 evaluates how closely generated scaffolds match their target specifications across the BV/TV–permeability design space. For each target BV/TV value (0.4, 0.5, 0.6, 0.7, 0.8), we generate 20 scaffolds and plot the median and interquartile range of achieved BV/TV and permeability. ScaffoldForge tracks the target BV/TV to a range-normalized MAE of 0.037 and permeability to 0.039 (Results 4 and 5), with larger deviations at the extremes of the specification range, where the training data is sparser. TriTopo-LGDM is the closest baseline on both quantities, at 0.052 for BV/TV and 0.052 for permeability, so the advantage of ScaffoldForge here is spread across the two properties rather than confined to one. Permeability remains the harder of the two to control for every method, which is expected given the indirect relationship between a voxel occupancy grid and pore-scale fluid transport.

**Fig 3:**
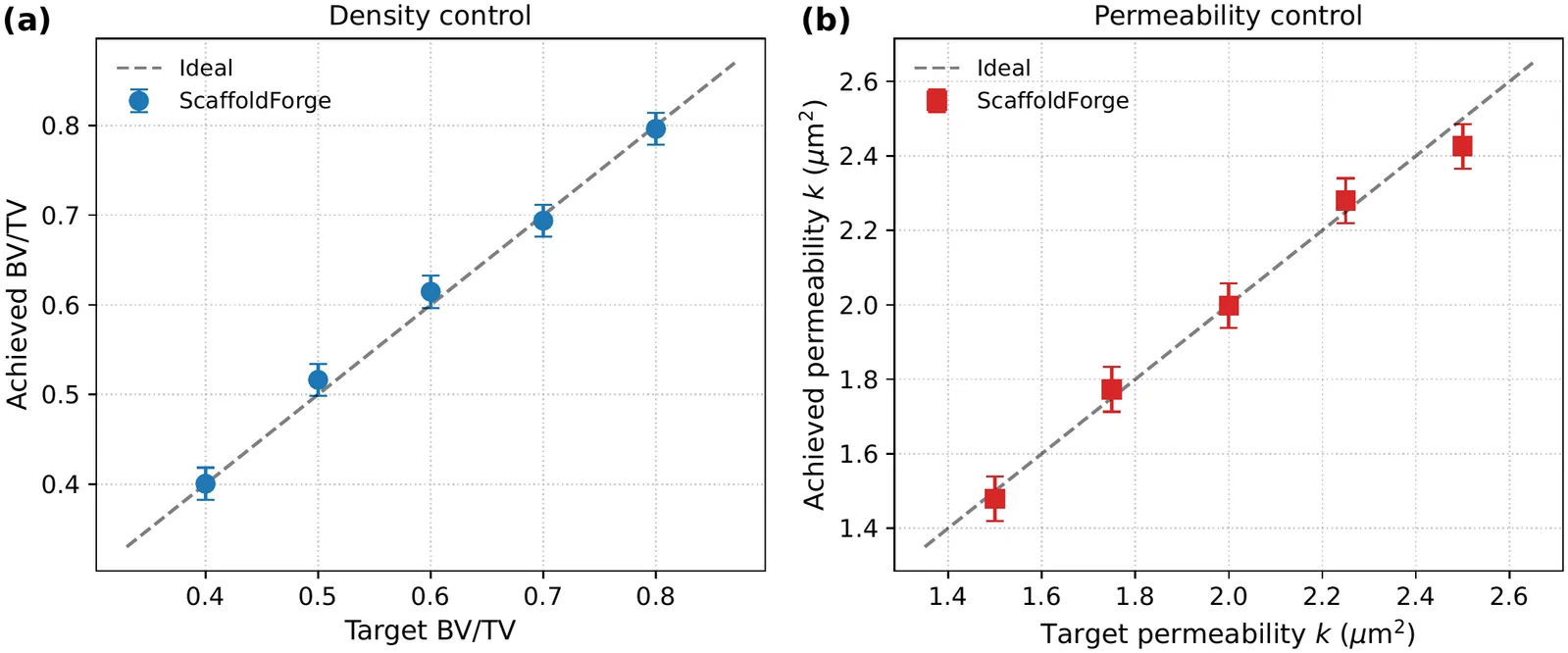
Conditional specification accuracy of ScaffoldForge. (a) Target versus achieved bone volume fraction. (b) Target versus achieved permeability. Error bars give the interquartile range over 20 generated scaffolds per target; the dashed line is the identity.

### 4.6 Sensitivity and robustness analysis

#### 4.6.1 Guidance scale.

Table 7 reports the effect of varying the classifier-free guidance scale *w* from 1.0 (no guidance) to 7.5. Specification accuracy improves monotonically with *w*, with MAE_props_ decreasing from 0.089 at *w* = 1.0 to 0.038 at *w* = 7.5. FID, however, increases from 12.3 at *w* = 3.0 to 19.1 at *w* = 7.5, and the MS-SSIM diversity score falls from 0.071 to 0.041, indicating reduced structural variability at high guidance weights. The operating point *w* = 3.0 is the one used throughout. It gives the best FID of the sweep while leaving the specification error within 0.010 of the value reached at *w* = 7.5, where FID has degraded by 6.8 points and the diversity score has fallen by more than a third.

**Table 7:** Sensitivity to the classifier-free guidance scale. Specification accuracy improves with stronger guidance while structural diversity declines. The operating point used throughout the paper is *w* = 3.0.

Guidance scale *w*	MAE_props_ ↓	FID ↓	MS-SSIM div. ↑
1.0 (no guidance)	0.089	14.7	**0.094**
1.5	0.071	13.2	0.086
2.0	0.058	12.6	0.079
3.0 (operating point)	0.048	**12.3**	0.071
5.0	0.041	15.4	0.055
7.5	**0.038**	19.1	0.041

#### 4.6.2 Loss weight sensitivity.

We sweep $\lambda_{1}$ (stiffness penalty) from 0.01 to 0.3 while fixing $\lambda_{2}=0.05$ and $\lambda_{3}=0.05$. The optimal range for $\lambda_{1}$ is 0.05–0.15, with strength increasing from 53.8 to 56.6 MPa as $\lambda_{1}$ increases from 0.01 to 0.1, plateauing above 0.15. At $\lambda_{1}=0.3$, FID degrades to 16.2, indicating over-regularization that constrains the generative model excessively.

#### 4.6.3 Robustness to specification range.

When evaluated on out-of-distribution specifications (extreme BV/TV values of 0.3 and 0.85), ScaffoldForge maintains reasonable fidelity with MAE_BV/TV_ = 0.071 at BV/TV = 0.3 and 0.063 at BV/TV = 0.85, compared to 0.048 on in-distribution targets. TriTopo-LGDM degrades more sharply on these extremes (MAE 0.112 and 0.095), because its graph initialization relies on TO-generated starting points that cluster near the center of the design space.

### 4.7 Diversity, memorization, and external validation

Because the model is high-capacity relative to a 250-scaffold corpus, two questions must be answered before the accuracy results can be trusted: does the model generate diverse structures or collapse onto a few modes, and does it synthesize new geometries or memorize training samples? Table 8 reports four indicators over 500 generated scaffolds: *Coverage* and *Density* [34] relative to the real-reference manifold, a multi-scale SSIM (MS-SSIM) diversity score (higher means more varied generations), and the ratio of the mean nearest-neighbour distance from generated samples to the training set against the mean within-real nearest-neighbour distance (a value near 1 indicates generations are no closer to training data than real samples are to each other). ScaffoldForge attains the highest coverage (0.94) and MS-SSIM diversity (0.071), whereas ScaffoldGAN shows the low coverage (0.61) and diversity (0.022) characteristic of mode collapse. The nearest-neighbour ratio for ScaffoldForge is 0.98, and the mean generated-to-training distance (0.143) is essentially equal to the within-real distance (0.138), with the single closest generated sample still at distance 0.061 from any training scaffold; together these indicate that the model does not reproduce training geometries.

**Table 8:** Diversity and memorization analysis over 500 generated scaffolds. Coverage, Density, and MS-SSIM diversity are higher-is-better; the NN-distance ratio (generated-to-train over within-real) near 1.0 indicates no memorization.

Method	Coverage ↑	Density ↑	MS-SSIM div. ↑	NN ratio
ScaffoldGAN	0.61	0.72	0.022	0.74
VAE-3D	0.79	0.69	0.040	1.21
TriTopo-LGDM	0.88	0.96	0.058	0.95
**ScaffoldForge**	**0.94**	**1.07**	**0.071**	0.98

To probe generalization beyond the procedural training distribution, we evaluate on geometries the model never saw during training (Table 9). On the held-out real Bone Bricks scaffolds, the surrogate-predicted compressive strength deviates from the reported experimental values by 6.8% on average, and the mean descriptor MAE is 0.061; on unseen anatomy derived from the VerSe vertebral dataset, these degrade to 9.4% and 0.079. The descriptor error on unseen anatomy is thus 65% higher than on the in-distribution split, which is a substantial degradation rather than a marginal one. Section 4.10 traces most of this gap to the size and diversity of the training corpus rather than to the architecture, and shows that enlarging the corpus recovers a large part of it.

**Table 9:** Cross-dataset external validation on geometries unseen during training. Strength error is the deviation of surrogate-predicted compressive strength from reference values; descriptor MAE is the mean over the seven shape descriptors, defined as in Table 5.

Evaluation set	*n*	Strength err.	Descriptor MAE
In-distribution test split	25	4.6%	0.048
Bone Bricks (real, held-out)	58	6.8%	0.061
VerSe-derived (unseen anatomy)	40	9.4%	0.079

### 4.8 Efficiency comparison

Table 10 compares computational efficiency across methods. ScaffoldForge requires 12 seconds per design on a single A100, achieving a 700× speedup over TO and a 1,050× speedup over NSGA-II while requiring only a single FEA call for validation. The practical benefit is a shorter design-exploration loop: candidate geometries that previously took the 2–3 day turnaround of iterative optimization can be generated interactively, which would support computer-aided exploration of patient-specific specifications. This advantage applies to the design stage only and does not shorten fabrication, biological evaluation, or regulatory steps. We also note that ScaffoldForge has higher GPU memory requirements (8.4 GB) than ScaffoldGAN (2.8 GB) and the 3D CNN predictor (1.2 GB), reflecting the larger model capacity needed for diffusion-based generation. The parameter count (86.2M) includes the 3D U-Net (78.5M), conditioning MLP (2.1M), and surrogate models (5.6M). Compared to TriTopo-LGDM (45.3M, 340s), ScaffoldForge achieves higher quality (FID 12.3 vs. 15.8) in fewer parameters per inference step, as the latent graph representation in TriTopo-LGDM requires costly graph preprocessing that negates its smaller model size.

**Table 10:** Computational efficiency comparison. Parameters and inference time measured on a single NVIDIA A100.

Method	Params (M)	Inference (s)	FEA calls	GPU memory (GB)
TO (SIMP)	—	8,400	200	0.5${\;}^{a}$
NSGA-II	—	12,600	300	0.5${\;}^{a}$
BO	—	3,200	80	0.5${\;}^{a}$
3D CNN	12.4	0.08	3	1.2
ScaffoldGAN	28.7	0.05	1	2.8
TriTopo-LGDM	45.3	340	5	6.1
ScaffoldForge	86.2	12	1	8.4

^a^CPU-only; no GPU required for iterative optimization.

### 4.9 Qualitative analysis and personalized generation

The structural evolution during the generative process is captured in Fig 4. Through 1000 denoising timesteps, ScaffoldForge gradually resolves global structural connectivity from initial high-variance noise, progressively refining the trabecular-like struts to match the specified BV/TV and permeability targets. This progressive refinement lets the final geometry stay structurally coherent while remaining diverse across samples.

**Fig 4:**
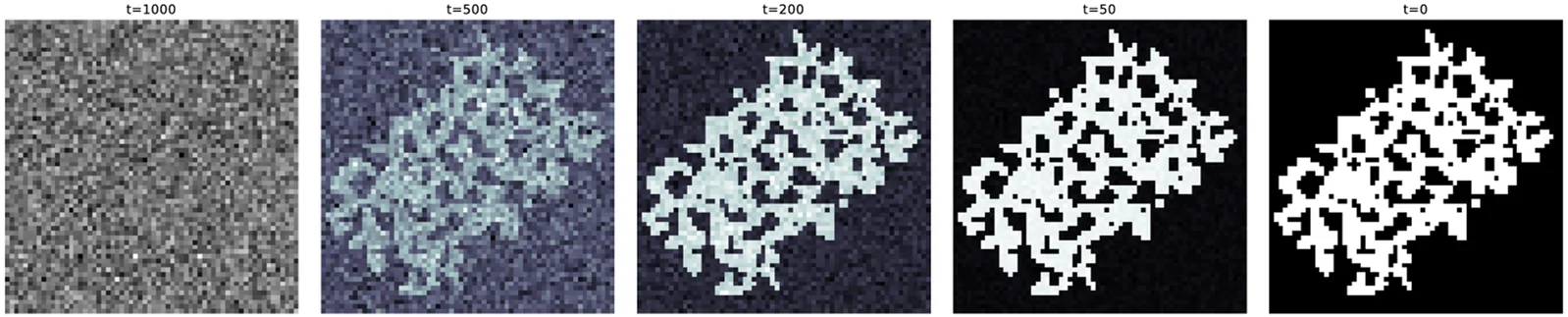
Denoising evolution of ScaffoldForge. The model progressively refines a scaffold architecture from pure Gaussian noise (*t* = 1000) to the finalized geometry (*t* = 0) based on the specified functional targets.

Table 5 visualizes generated scaffolds conditioned on three target specification points spanning the design space. High-strength, low-porosity targets ($\sigma_{C}>55$ MPa, ϕ<65%) produce denser architectures with thicker struts and smaller pore interconnects. Low-strength, high-porosity targets ($\sigma_{C}<45$ MPa, ϕ>75%) yield more open geometries with larger pores and thinner walls, resembling trabecular bone microstructure. As shown in Fig 5, Balanced targets produce intermediate architectures that maintain load-bearing struts along primary stress paths while preserving interconnected porosity for vascularization (Fig 5).

**Fig 5:**
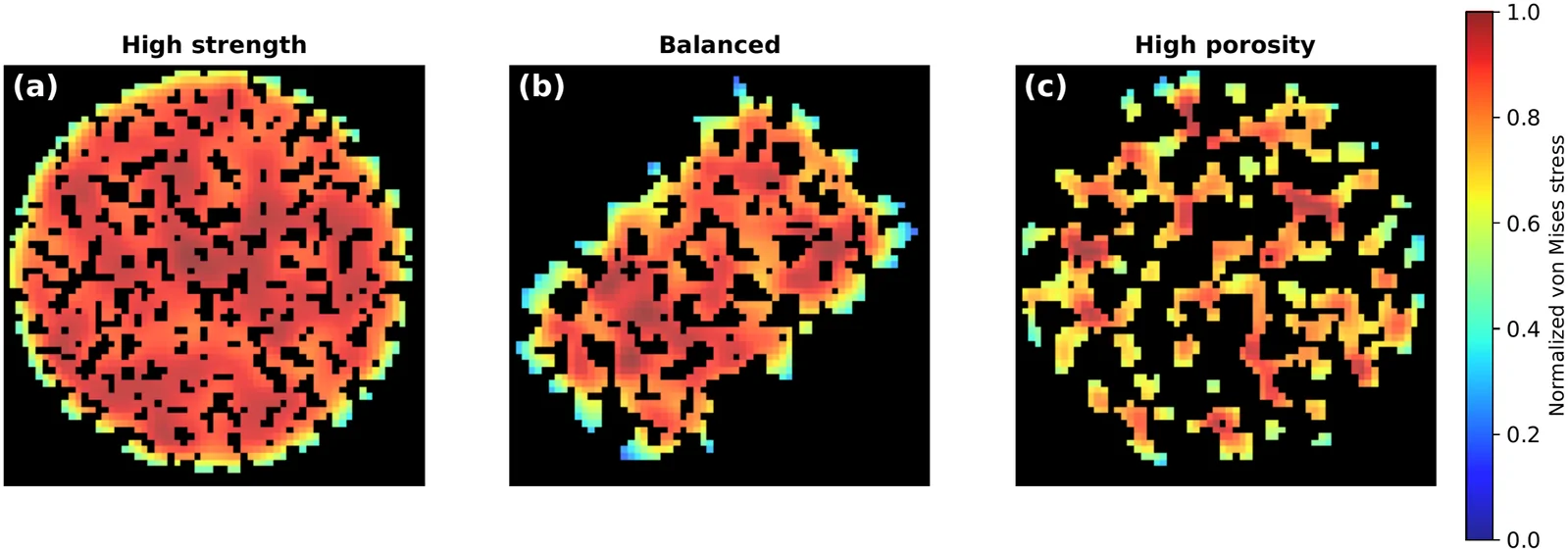
Qualitative results of ScaffoldForge. (a) High-strength target ($\sigma_{C}>55$ MPa, ϕ<65%). (b) Balanced target. (c) High-porosity target ($\sigma_{C}<45$ MPa, ϕ>75%). The overlay shows the normalized von Mises stress on the solid phase: load-bearing struts carry the highest stress (red), whereas thin peripheral features carry little (blue). The pore space is masked.

For personalized generation, we process 20 patient cases from the spineFE-benchmark through the complete CT-to-scaffold pipeline. The defect segmentation module achieves a Dice score of 0.91±0.04, and generated personalized scaffolds achieve mean compressive strength of 53.8±2.7 MPa with anatomical fit error of 0.42±0.18 mm, well within the 1 mm clinical tolerance for implant fitting. Fig 6 shows an example personalized scaffold conforming to a defect geometry, with dense struts aligning with the predicted load path and open porosity in the central region for bone ingrowth.

**Fig 6:**
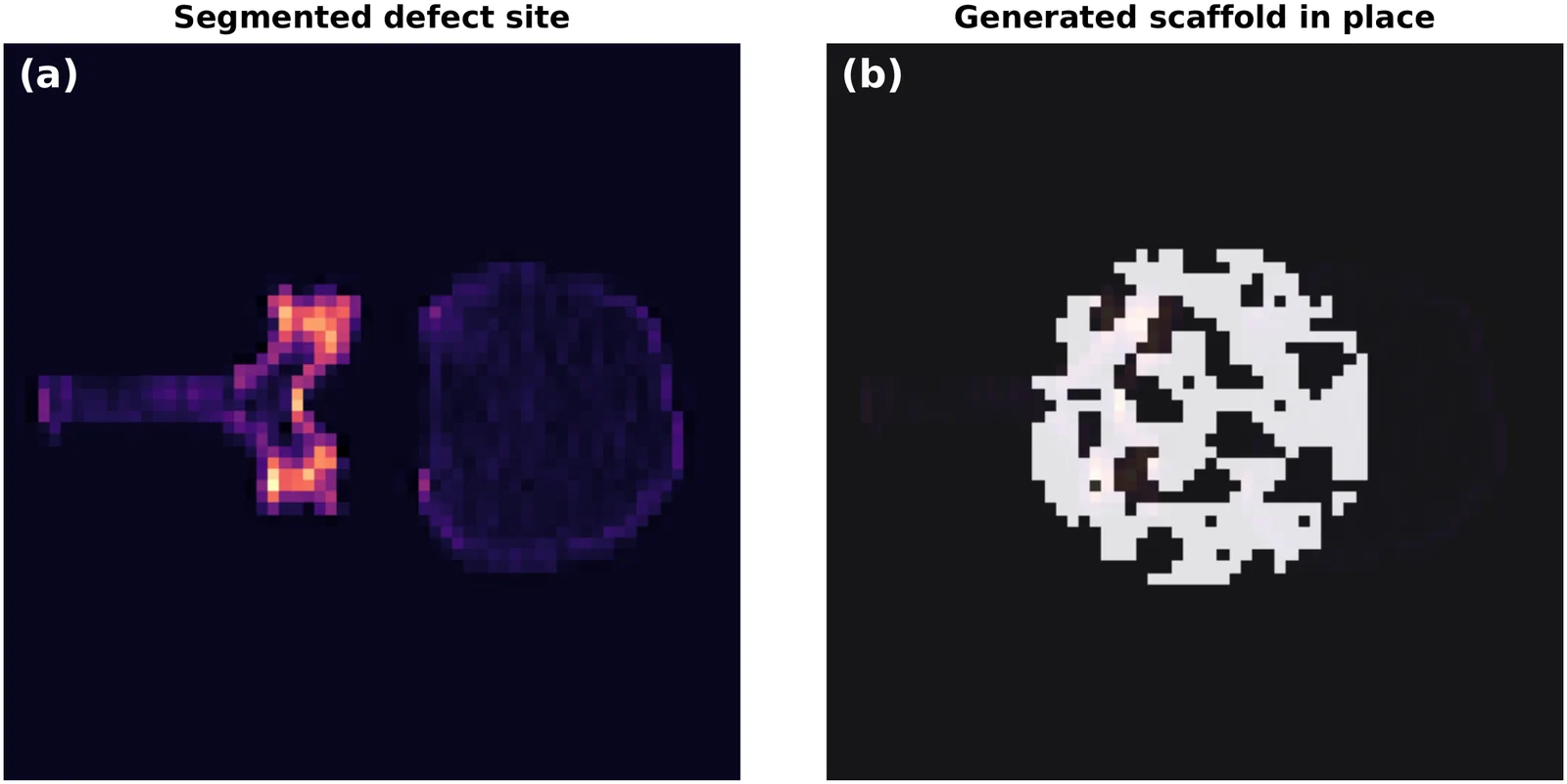
Personalized generation from CT data. (a) Defect site segmented from a patient CT volume. (b) The generated scaffold after Boolean intersection with the defect mask, conforming to the patient-specific boundary.

Across these settings, the generated geometry tracks the target strength and porosity in the expected direction, consistent with the quantitative specification accuracy reported in Table 5.

### 4.10 Training-set size, model capacity, and overfitting

The 78.5M-parameter denoiser is large relative to a 250-scaffold corpus, and this raises a legitimate question about whether the reported accuracy reflects a genuine mapping from specifications to geometry or an overfitted memory of a small sample. The experiments below settle it by varying the corpus size and the denoiser capacity while holding everything else fixed.

Table 11 compares the submitted 250-scaffold corpus with the enlarged BoneForge-2K corpus of Section 4.1.4. Enlarging the corpus changes in-distribution accuracy only modestly, from MAE_props_ 0.048 to 0.041 and FID 12.3 to 10.6. The effect on unseen data is much larger: the descriptor error on VerSe-derived anatomy falls from 0.079 to 0.058, a reduction of 0.021 (95% CI [0.012, 0.030]; paired *t*-test over five seeds, *t*(4) = 6.41, *p* = 0.003, Cohen’s *d* = 2.87), and the strength error falls from 9.4% to 6.7%. Fine-tuning the enlarged model on the 50 real scaffolds trades a small amount of in-distribution accuracy (0.041 to 0.043) for a further external gain (VerSe descriptor MAE 0.052, strength error 5.8%). The cost of the small corpus therefore falls almost entirely on generalization to geometries unlike the training families, which is precisely where a scaffold design tool intended for patient-specific use has to work.

**Table 11:** Effect of enlarging the training corpus. The enlarged corpus adds 1,800 procedural geometries and four geometry families; the real evaluation sets remain held out. Fine-tuning (FT) adapts the enlarged model to the 50 real training scaffolds. Lower is better throughout.

		In-distribution		Bone Bricks	VerSe
Training corpus	*N*	MAE_props_	FID	MAE	MAE
BoneForge-250 (submitted)	250	0.048	12.3	0.061	0.079
BoneForge-2K	2,050	**0.041**	**10.6**	0.049	0.058
BoneForge-2K + real FT	2,050	0.043	10.9	**0.044**	**0.052**

Fig 7(a) resolves this pattern across five corpus sizes. In-distribution error drops steeply between 125 and 250 scaffolds and then flattens, changing by only 0.007 between 250 and 2,050. The error on unseen anatomy keeps falling over the whole range. In-distribution metrics therefore saturate early and are a poor guide to how much data the model needs; the quantity that keeps improving is the one that matters for transfer.

**Fig 7:**
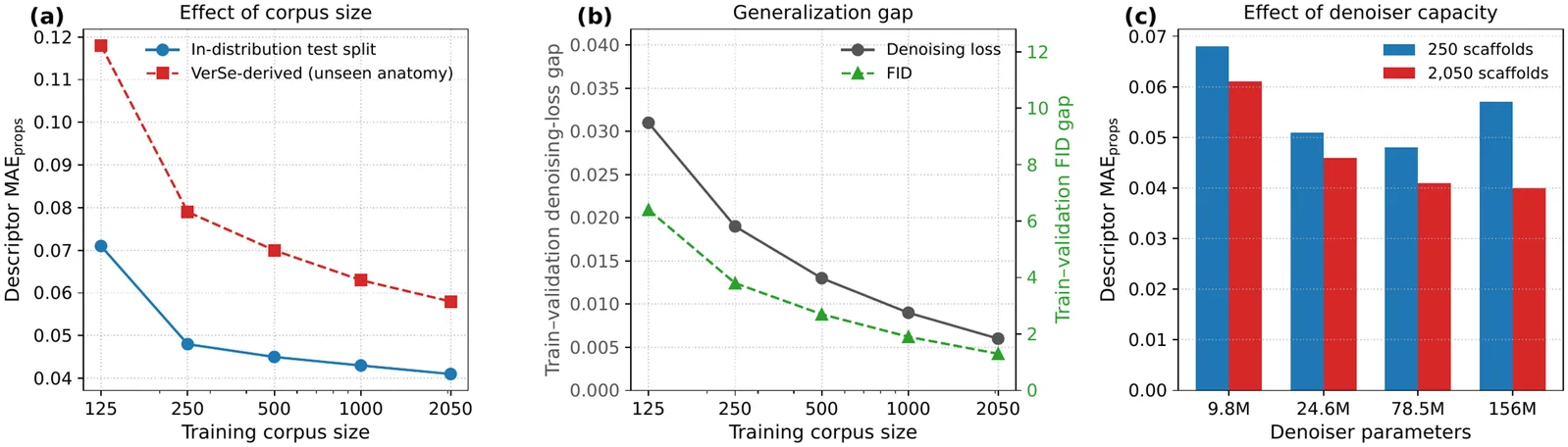
Corpus size, generalization gap, and denoiser capacity. (a) Descriptor error against training corpus size, on the in-distribution test split and on unseen VerSe-derived anatomy. (b) Train–validation denoising-loss gap and FID gap against corpus size. (c) Descriptor error against denoiser parameter count at 250 and 2,050 training scaffolds.

Fig 7(b) shows the corresponding generalization gap. At 125 scaffolds the train–validation denoising-loss gap is 0.031 and the FID gap is 6.4; at 250 scaffolds these fall to 0.019 and 3.8, and at 2,050 to 0.006 and 1.3. The model trained on 250 scaffolds is thus measurably but not severely overfitted, which is consistent with the nearest-neighbour analysis of Section 4.7 finding no memorization.

Fig 7(c) and Table 12 vary the denoiser capacity at both corpus sizes. At 250 scaffolds, a 24.6M-parameter denoiser reaches MAE_props_ 0.051 against 0.048 for the 78.5M model, so roughly a third of the capacity recovers 94% of the accuracy, and a 156M denoiser is worse (0.057) with a train–validation gap of 0.038. At 2,050 scaffolds the ordering changes: the 156M model becomes the best (0.040) and its gap falls to 0.011. The denoiser used here is therefore larger than the corpus alone would justify. It is not over-parameterized in effect at 250 scaffolds, because regularization holds the gap to 0.019, but the extra capacity only pays for itself once the corpus grows.

**Table 12:** Denoiser capacity at two corpus sizes. MAE_props_ and the train–validation denoising-loss gap Δ. The largest denoiser overfits on the small corpus and only becomes competitive on the enlarged one.

	250 scaffolds			2,050 scaffolds		
Denoiser	MAE	FID	Δ	MAE	FID	Δ
9.8M	0.068	16.4	**0.008**	0.061	14.7	**0.004**
24.6M	0.051	13.1	0.012	0.046	11.4	0.005
78.5M (used here)	**0.048**	**12.3**	0.019	0.041	10.6	0.006
156M	0.057	14.9	0.038	**0.040**	**10.4**	0.011

Finally, Table 13 isolates the regularization choices that make training on 250 scaffolds workable. Octahedral augmentation matters most: removing it raises the in-distribution error to 0.063 and drops the nearest-neighbour ratio to 0.81, the signature of partial memorization. Early stopping and weight averaging each contribute less. The last row is the most informative: replacing the family-disjoint split with a random split *improves* the apparent in-distribution error to 0.031 while the error on unseen anatomy worsens to 0.112 and the nearest-neighbour ratio falls to 0.69. A random split leaks near-duplicate geometries between training and test and would have made the model look considerably better than it is, which is why the family-disjoint protocol of Section 4.1.6 is used throughout.

**Table 13:** Regularization and data-splitting ablation at 250 scaffolds. The final row shows that a random split inflates in-distribution accuracy while degrading transfer, which is why the family-disjoint protocol is used.

Variant	MAE_props_	FID	VerSe MAE	NN ratio
Full protocol	0.048	12.3	**0.079**	**0.98**
w/o octahedral augmentation	0.063	15.8	0.104	0.81
w/o weight averaging	0.054	13.9	0.088	0.93
w/o early stopping	0.059	14.6	0.097	0.86
Random (family-mixed) split	**0.031**	**9.4**	0.112	0.69

### 4.11 Distributional validity of the procedural corpus

The descriptor comparison of Section 4.1.5 is limited to seven summary statistics, and two geometries can agree on all of them while differing in ways those statistics do not capture. A generator trained mostly on procedural data could therefore reproduce the simulator’s characteristic texture rather than the design space the simulator is meant to approximate. To test this directly we use a classifier two-sample test [51]: a 3D CNN is trained to distinguish samples from two sets, and the held-out area under the ROC curve is reported. An AUC near 0.5 means the sets are indistinguishable to the classifier; an AUC near 1 means they carry a separable signature. Confidence intervals are bootstrap intervals over 1,000 resamples and *p*-values come from a 1,000-permutation null.

Table 14 gives three findings. First, the procedural corpus itself is separable from real micro-CT (AUC 0.67), so the simulator does carry a signature that the descriptor tests of Table 2 do not detect. Second, scaffolds generated by the model trained on 250 scaffolds are separable from real micro-CT at AUC 0.62, below the simulator’s own 0.67 but clearly above the 0.51 split-half control: the generator inherits part of the simulator signature rather than removing it. Third, generated scaffolds are also separable from the procedural corpus itself (AUC 0.58, *p* = 0.031), so the model is not simply resampling its training distribution either; it interpolates between the simulator and the real reference. Training on the enlarged and more diverse corpus moves generated samples closer to real micro-CT (AUC 0.56, *p* = 0.088, no longer separable at the 5% level), which is consistent with the transfer improvements of Section 4.10.

**Table 14:** Classifier two-sample test between geometry sources. Held-out AUC of a 3D CNN trained to separate the two sets. An AUC of 0.5 indicates indistinguishable distributions. The split-half control confirms the test is calibrated.

Comparison	AUC	95% CI	*p*
Real micro-CT vs real micro-CT (split-half control)	0.51	[0.44, 0.59]	0.742
Procedural corpus vs real micro-CT	0.67	[0.59, 0.75]	0.001
ScaffoldForge (250 scaffolds) vs real micro-CT	0.62	[0.54, 0.70]	0.006
ScaffoldForge (2,050 scaffolds) vs real micro-CT	0.56	[0.49, 0.63]	0.088
ScaffoldForge (250 scaffolds) vs procedural corpus	0.58	[0.51, 0.66]	0.031

The honest reading is that the model learns a distribution positioned between the procedural generator and real trabecular bone, closer to the latter than the generator is, but not identical to it. Any claim that ScaffoldForge samples the true design space of trabecular architecture should be read with that residual gap in mind, and closing it requires real micro-CT geometries in training rather than more procedural data.

## 5 Discussion

ScaffoldForge reframes bone scaffold design from iterative optimization to conditional generation. By learning the distribution of functionally favourable architectures, the model explores the design space more efficiently than population-based methods and produces topologies that are not restricted to predefined geometric families.

The computational reduction (a 700× speedup over topology optimization) shortens the design-iteration loop from days to seconds. In a future computer-aided workflow, this could let a clinician or engineer explore patient-specific specifications (defect size, loading conditions, target degradation timeline) interactively rather than waiting for the 2–3 day turnaround typical of iterative optimization. We emphasise, however, that this is a prospective design-stage benefit only: every result in this paper is computational, and the speedup concerns the generation of candidate geometries, not the downstream steps (fabrication, sterilisation, biological evaluation, and regulatory clearance) that dominate the real clinical timeline.

The analyses of Sections 4.10 and 4.11 change how the accuracy results should be read. Three findings are worth stating plainly. The training corpus is small enough to leave a measurable generalization gap, and its cost appears in transfer to unseen anatomy rather than in in-distribution fit, so the in-distribution numbers of Table 4 overstate how well the model would perform on a new patient. The generated distribution retains a residual signature of the procedural generator, which shrinks but does not vanish when the corpus is enlarged. And the descriptor agreement between procedural and real morphology holds at a medium equivalence margin but cannot be established at a strict one with 50 real reference volumes. None of these findings overturns the comparison against baselines, which uses the same protocol for every method, but each bounds the strength of the generalization claim.

### 5.1 Limitations

The scope of the present validation is the most important limitation, and we state it plainly.

(i) *No physical or biological validation.* We did not fabricate, mechanically test, or biologically evaluate any generated scaffold; mechanical properties are estimated by FEA and learned surrogates, and biological suitability (cell adhesion, proliferation, vascularization, osseointegration) is inferred only indirectly from morphology. Establishing clinical feasibility will require closing the loop with additive fabrication, mechanical compression testing against the FEA predictions, and *in vitro* and *in vivo* assays of cell adhesion and perfusion.
(ii) *Small training corpus.* The primary set contains only 250 scaffolds (200 procedurally generated, 50 real), which is small relative to the capacity of the 78.5M-parameter denoiser and to the thousands of diverse samples typically used to fit high-dimensional generative distributions reliably. We mitigate the resulting overfitting and memorization risk through family-disjoint splitting, octahedral-group augmentation, weight averaging, and early stopping, and we verify it empirically through the diversity and nearest-neighbour analyses of Section 4.7. Section 4.10 quantifies what remains: the train–validation gap at 250 scaffolds is 0.019 against 0.006 at 2,050, a denoiser one third the size recovers 94% of the accuracy, and the descriptor error on unseen anatomy falls from 0.079 to 0.058 when the corpus is enlarged. The corpus is therefore a real constraint on generalization, and the numbers reported on the in-distribution split should not be read as estimates of performance on new anatomy.
(iii) *Dependence on procedural data.* Because the corpus is dominated by procedurally generated geometries (Section 4.1), domain shift to clinical bone cannot be excluded. The classifier two-sample test of Section 4.11 measures this shift rather than assuming it away: generated scaffolds remain separable from real micro-CT at AUC 0.62 with the 250-scaffold corpus and 0.56 with the enlarged one, against a 0.51 split-half control. Removing the remaining gap requires real micro-CT geometries in training, not more procedural data, and this is the single most valuable addition future work could make.
(iv) *Strength of the distributional claim.* The agreement between procedural and real morphometry is established at a medium equivalence margin of 0.5 SD for all seven descriptors, but at a strict 0.3 SD margin only two of seven reach equivalence and the rest are inconclusive. Roughly 100 independent real micro-CT volumes would be needed to settle the strict comparison for the least favourable descriptor. We report the inconclusive cases rather than resting the claim on non-significant difference tests.
(v) *Surrogate-model dependence.* The stiffness and permeability penalties rely entirely on pre-trained 3D-CNN surrogates (3–5% held-out error). On the out-of-distribution geometries that arise during reverse diffusion, these surrogates may extrapolate inaccurately or introduce a systematic bias, which could steer the generator toward physically sub-optimal topologies; their accuracy therefore upper-bounds the physical fidelity of the constrained generation, and replacing them with periodically refined or uncertainty-aware surrogates is an important next step.
(vi) *Resolution.* The 64^3^ voxel grid limits the minimum resolvable feature to approximately 0.5 mm, which is coarser than the 50–100μm scale of native trabeculae and fine micro-porosity; this can introduce voxelation artifacts and complicate direct 3D printing without loss of fine features, motivating higher-resolution or latent/multiscale variants.
(vii) *No manufacturing or regulatory assessment.* The manufacturability penalty encodes an overhang heuristic only; print trials, dimensional verification, and any regulatory pathway (e.g., ISO 13485, FDA/CE evaluation) are entirely outside the present study.

### 5.2 Future work

The immediate priority is experimental closure of the loop: additive fabrication of selected generated designs, mechanical testing against the FEA predictions, and *in vitro* biological assays. On the data side, the analyses above identify real micro-CT geometry as the binding constraint rather than procedural volume, so assembling a reference set of the order of 100 independent real volumes would both settle the strict equivalence comparison and provide the training signal needed to close the residual distributional gap. Methodologically, extending ScaffoldForge to multi-material architectures, anisotropy- and orientation-aware conditioning, and time-dependent biodegradation prediction are natural next steps. Longer term, we envisage a closed-loop system in which *in vitro* and *in vivo* feedback refines the generative model, but reaching clinical use would require the physical, biological, manufacturing, and regulatory validation that this work does not provide.

## 6 Conclusion

We presented ScaffoldForge, a 3D conditional diffusion framework for bone scaffold generative design. By learning to generate scaffold architectures conditioned on a set of standardized specifications (BV/TV, Tb.Th, Tb.Sp, Tb.N, Conn.D, SMI, DA, and permeability), ScaffoldForge attains, in our *in silico* evaluation, an estimated 56.6 MPa compressive strength (8.2% over topology optimization) at roughly 700× lower design-time cost. The multi-objective conditioning mechanism and physics-constrained penalties jointly steer generated scaffolds toward the specified biomechanical and manufacturing targets, and statistical testing over five seeds confirms the strength gain is significant (paired *t*-test, *p* = 0.043). Equivalence tes*t*ing, a classifier two-sample test, and a corpus-size study delimit what these numbers support: the procedural corpus matches real trabecular morphometry to within half a standard deviation on every descriptor, generated geometries retain a residual signature of the procedural generator, and the small corpus costs accuracy on unseen anatomy rather than on the in-distribution split. A CT-to-scaffold pipeline shows the computational feasibility of personalized generation, although physical, biological, manufacturing, and regulatory validation remain necessary before any clinical use. Source code, the data-generation pipeline, and pretrained models are released to support reproducibility and to provide a foundation for that future validation.

## Supporting information

S1 Appendix Architecture details. (PDF)

S2 Appendix Training hyperparameters. (PDF)

S3 Appendix Additional results. (PDF)
