# Supplementary material for: ScaffoldForge: Bioinspired generative design of 3D-printed bone scaffolds via conditional diffusion for large segmental bone defect reconstruction
Source: PLoS One. 2026 Oct 28;21(10):e0361041. doi: 10.1371/journal.pone.0361041 (PMC13716220; doi:10.1371/journal.pone.0361041)
Supplement: Supplementary file 1 [file pone.0361041.s001.pdf]

Supporting information

S1 Appendix. Architecture details. The denoising network is a 3D U-Net with four resolution levels operating on 64^3 voxel grids. The encoder channel widths are $\{64, 128, 256, 512\}$, with two residual blocks per level and strided convolutions for downsampling; the decoder mirrors the encoder with transposed convolutions and skip connections. Each residual block applies group normalization, a SiLU nonlinearity, and an AdaIN modulation layer (Eq (5)) whose scale and shift are produced by per-level linear projections of the condition embedding $\mathbf{z} \in \mathbb{R}^{256}$. Multi-head self-attention (4 heads) is inserted at the 16^3 and 8^3 resolutions. The timestep t is encoded with a sinusoidal embedding followed by a two-layer MLP and added to the block features. The condition encoder MLP_ϕ maps the eight normalized descriptors through two hidden layers of width 512 (SiLU) to the 256-dimensional embedding; a learnable null token implements the classifier-free dropout. The two surrogate models $\hat{\sigma}_C$ and $\hat{k}$ are separate 3D CNNs with four convolutional blocks ($\{32, 64, 128, 256\}$ channels) followed by global average pooling and a two-layer regression head. The denoiser, condition encoder, and surrogates contain approximately 78.5M, 2.1M, and 5.6M parameters, respectively. The capacity variants of Table 12 scale the channel widths to $\{32, 64, 128, 256\}$ (9.8M), $\{48, 96, 192, 384\}$ (24.6M), and $\{96, 192, 384, 768\}$ (156M), leaving depth and attention placement unchanged.
