# Supplementary material for: ScaffoldForge: Bioinspired generative design of 3D-printed bone scaffolds via conditional diffusion for large segmental bone defect reconstruction
Source: PLoS One. 2026 Oct 28;21(10):e0361041. doi: 10.1371/journal.pone.0361041 (PMC13716220; doi:10.1371/journal.pone.0361041)
Supplement: Supplementary file 2 [file pone.0361041.s002.pdf]

Supporting information

S2 Appendix. Training hyperparameters. Table 15 lists the complete training configuration. All values are shared across the five random seeds used for the reported means.

Table 15. Training and inference hyperparameters for ScaffoldForge.

Hyperparameter	Value
Voxel resolution	$64 \times 64 \times 64$
U-Net channels	$\{64, 128, 256, 512\}$
Self-attention resolutions	16^3, 8^3
Condition embedding dim. d	256
Diffusion steps T	1000
Noise schedule	cosine
Optimizer	Adam (β_1=0.9, β_2=0.999)
Learning rate	2×10^{-4}
LR schedule	500-step warmup, cosine decay
Batch size	16
Epochs	200
EMA decay	0.9999
CFG dropout p_{drop}	0.1
Guidance scale w	3.0
Penalty weights $(\lambda_1, \lambda_2, \lambda_3)$	$(0.1, 0.05, 0.05)$
Penalty cutoff $t_{\max}$	300
Occupancy relaxation (κ, τ)	$(8, 0.5)$
Overhang sharpness α	10
Hardware	2× NVIDIA A100 80GB
