# Supplementary material for: ScaffoldForge: Bioinspired generative design of 3D-printed bone scaffolds via conditional diffusion for large segmental bone defect reconstruction
Source: PLoS One. 2026 Oct 28;21(10):e0361041. doi: 10.1371/journal.pone.0361041 (PMC13716220; doi:10.1371/journal.pone.0361041)
Supplement: Supplementary file 3 [file pone.0361041.s003.pdf]

Supporting information

S3 Appendix. Additional results. Table 5 reports the per-descriptor specification accuracy that supports the aggregate $\mathrm{MAE}_{\mathrm{props}}$ in Table 4, and Tables 2, 8, and 9 provide the morphometric, diversity/memorization, and cross-dataset external-validation analyses discussed in Section 4.1 and Section 4.7. Across the three evaluation sets, performance degrades monotonically with distance from the training distribution (in-distribution, real held-out, unseen anatomy), which is the expected behaviour for a model trained predominantly on procedural geometries. Tables 11, 12, 13 and 14 report the corpus-size, capacity, regularization, and distributional analyses of Sections 4.10 and 4.11. The data-generation pipeline, trained models, and the script that produces these quantitative summaries are released in the public repository to support full reproduction.
